# Hybrid species evolution between cryptic and sympatric weevil species

**DOI:** 10.1098/rspb.2026.1745

**Published:** 2026-10-28

**Authors:** Víctor Noguerales, Marius H. Eisele, Sophia L. Gray, Sean Stankowski, Brent C. Emerson

**Affiliations:** ^1^ Institute of Natural Products and Agrobiology (IPNA-CSIC), San Cristóbal de La Laguna, Spain; ^2^ Instituto de Investigación en Recursos Cinegéticos (IREC - CSIC, UCLM, JCCM), Ciudad Real, Spain; ^3^ Department of Ecology and Evolution, University of Sussex, Brighton, UK; ^4^ Department of Genetics, Evolution, and Environment, University College London, London, UK

**Keywords:** coleoptera, insect, hybridization, homoploid hybrid speciation, reproductive isolation, speciation

## Abstract

Hybridization may generate a variety of evolutionary outcomes, including novel hybrid species without a change in ploidy. Within this process, referred to as homoploid hybrid speciation (HHS), selection on recombinant traits is often invoked as the driver of reproductive isolation (RI) between hybrid and parental species. However, our understanding of the temporal, geographical and phenotypic dimensions within which hybrid populations evolve RI against parental species is still limited. Here, we describe speciation following hybridization within a beetle species complex that presents no obvious phenotypical or ecological differences among hybrid and parental species, and where all occur sympatrically. Demographic modelling supports hybrid species origin, where the hybrid lineage originated from historical admixture between parental lineages. Genome-wide estimates of heterozygosity and individual-based simulations of hybrid species origin provide further support for an ancient, rather than recent origin of the hybrid species. We use geographical patterns of genomic relatedness between hybrids and parentals to identify the probable origin of the hybrid species within a hybrid swarm that was geographically isolated from both parental species. We then discuss our results within the context of HHS theory to highlight predictions for an HHS origin that can be tested when an appropriate reference genome becomes available.

## 1. Introduction

Homoploid hybrid speciation (HHS) appears to be relatively rare in nature, but evidence for it is increasingly being detected through analyses of genome-wide nuclear genetic variation that reveal mosaic genomes which are assigned to more than one parental species [1,2]. HHS, in its most basic definition, is the formation of new species that are reproductively isolated from the parental species from which they have been derived through hybridization, and where ploidy is the same as that of the parental species [3,4]. Beyond this basic definition, there are divergent opinions about precisely what defines a hybrid species. Perhaps the most stringent definition is that of Schumer *et al.* [5], who suggest that hybrid species should meet three criteria. The first is that there should be strong reproductive isolation (RI) between parental and hybrid species. The second is that hybridization should be evidenced by genetic data. The third is that RI should be directly derived from hybridization itself. This definition seeks to identify hybridization-derived isolating barriers, such as assortative mating, and ecological or genetic incompatibilities, as opposed to those that arise after hybridization owing to sorting of ancestral variants or new mutations [6]. By contrast, Nieto Feliner *et al.* [3] have argued that by being overly restrictive, the definition of Schumer *et al.* [5] may underestimate the importance of hybridization in generating new species, because few species will satisfy all three criteria. This is particularly true for the third criterion, that RI should be derived from hybridization itself, as it is difficult to test within hybrid lineages of older origin [7,8], although in some cases this can be addressed with whole-genome data when candidate genes associated with RI can be identified [9].

Because strong RI is typically polygenic, Long & Rieseberg [9] argue that HHS may be more protracted, and thus more common than has been suggested by conceptual arguments and theoretical studies. While the criteria of Schumer *et al.* [5] imply hybridization itself to be important for the establishment of initial reproductive barriers against parental species, Long & Rieseberg [9] propose an extended model. They point out that many models of hybrid speciation involve the sorting of parental species barriers, such that hybrid lineages will initially be less strongly isolated from the parental species than the parental species are to each other. Thus, as admixed populations form and diverge, even in the face of initially maladaptive or neutral admixture, they may at some point lead to HHS. This polygenic model is still consistent with the model of Schumer *et al.* [5,10], but with protracted rather than instantaneous (or close to instantaneous) RI [9].

Hybridization can thus be considered not only a fast-acting driver for speciation, through selection acting on hybrid phenotypes or adaptively evolving Bateson–Dobzhansky–Muller (BDM) genetic incompatibilities, but also a slower acting facilitator of speciation where selection acts on neutrally evolving BDM loci. We favour this broader model of HHS, as it encompasses both the definition of Schumer *et al.* [5], where hybridization may act as the driving agent of RI, while also addressing the concerns of Nieto Feliner *et al.* [3], where hybridization may play a less immediate role, but nevertheless facilitate the evolution of RI. However, challenges to demonstrate that hybridization led to the emergence of RI remain. Experimental crosses and quantitative trait locus (QTL) analysis are powerful approaches [5], but are limited to species that are amenable to such experimentation. Candidate gene approaches have also been used, although convincing association with the evolution of RI requires functional validation, genome-wide association studies or QTL analysis [9]. In any case, such approaches are reliant on trait variation between parental species.

Given that hybrids are considered to probably be ecologically intermediate [11], and that overlapping mating signals and preferences of hybrid populations with parental species are also likely to limit hybrid species formation, HHS has been considered to be infrequent [5]. However, two phenomena may counter this. First, hybrid speciation is necessarily set within a history of speciation with gene flow that should intuitively act against the evolution of RI within a hybrid lineage. However, there is empirical support for a Goldilocks zone of divergence [12,13] that may be optimal for hybrid speciation to occur. The Goldilocks zone was originally described in the context of specific linkage architectures and intermediate recombination rates that might favour hybrid speciation [12,14], but has also been applied by Comeault & Matute [13] to demonstrate that assortative mating is more pronounced within hybrid lineages at intermediate levels of divergence between hybridizing parental species. Second, the emergence of transgressive phenotypes (i.e. falling outside the range of parental variation) may favour the establishment of reproductive barriers between hybrid populations and their parental species [15,16]. Indeed, most examples attributed to HHS involve parental species characterized by obvious ecologically or behaviourally relevant trait divergence [17–19], within which hybridization may give rise to transgressive phenotypes.

Here we present a unique case of speciation following hybridization, wherein the hybrid species and both parental species all occur sympatrically within a geographically limited area, in the absence of obvious ecological divergence among the parental and hybrid species. *Silvacalles instabilis* (Wollaston, 1864) is a species of hidden snout weevil (Coleoptera: Curculionidae), endemic to the Canary Islands, restricted to the range-limited cloud forest formations within the islands of Tenerife and Gran Canaria. Structured sampling on the island of Tenerife has found *S. instabilis* to be limited to above-ground vegetation [20] and associated with a variety of tree species (e.g. *Laurus novocanariensis, Picconia excelsa, Persea indica, Myrica faya* and *Ilex platyphylla*), where it probably feeds on dead or dying wood and bark, as is characteristic of many species within the subfamily Cryptorhynchinae. The species is small-bodied (less than 5 mm) and lacks wings, with its poor dispersal ability associated with strong geographical structuring of genetic variation over limited spatial scales [20]. *Silvacalles instablis* is 1 of 16 species of Coleoptera that have previously been characterized with genome-wide nuclear data across the cloud forest of the Anaga peninsula of Tenerife [20]. In the case of *S. instabilis*, in addition to the genome-wide nuclear data used in Salces-Castellano *et al.* [20], several divergent nuclear genotypes, indicative of a probable second species, were originally excluded from analysis and assumed to belong to the sister species *Silvacalles nubilosus* (Wollaston, 1864). However, a posterior revision of this material using a reference library of mitochondrial barcodes for the weevils of the Macaronesian region [21] unequivocally assigns the divergent genotypes to *S. instabilis*.

We first clarify species boundaries and taxonomic assignment with a broader sampling of *S. instabilis*, together with individuals of *S. nubilosus*. We then assess population structuring of nuclear genomic variation within *S. instabilis*. We finally use the genome-wide nuclear data for a demographic modelling approach to understand the dynamics of gene flow and admixture within the speciation history of the *S. instabilis* complex. Our results provide unequivocal support for an initial cladogenetic event that underpins the origin of two species, followed by admixture between these two parental lineages giving rise to a third hybrid species. We discuss the well-documented spatio-temporal dynamics of species ranges within the focal habitat, and the potential for these to have catalysed the formation of a hybrid species. We then discuss our results in the context of HHS theory, and the potential for hybridization to have directly contributed to the evolution of RI between the hybrid and parental species.

## 2. Material and methods

### (a) Sample collection

Previous sampling of *S. instabilis* by Salces-Castellano *et al.* [20] was enhanced with 11 additional sites along the Anaga peninsula [22], yielding a total of 106 specimens from 21 sampling sites (figure 1; supplementary material, figure S1 and table S1). The supplementary material, methods S1 provides further details on sampling.

**Figure 1.**
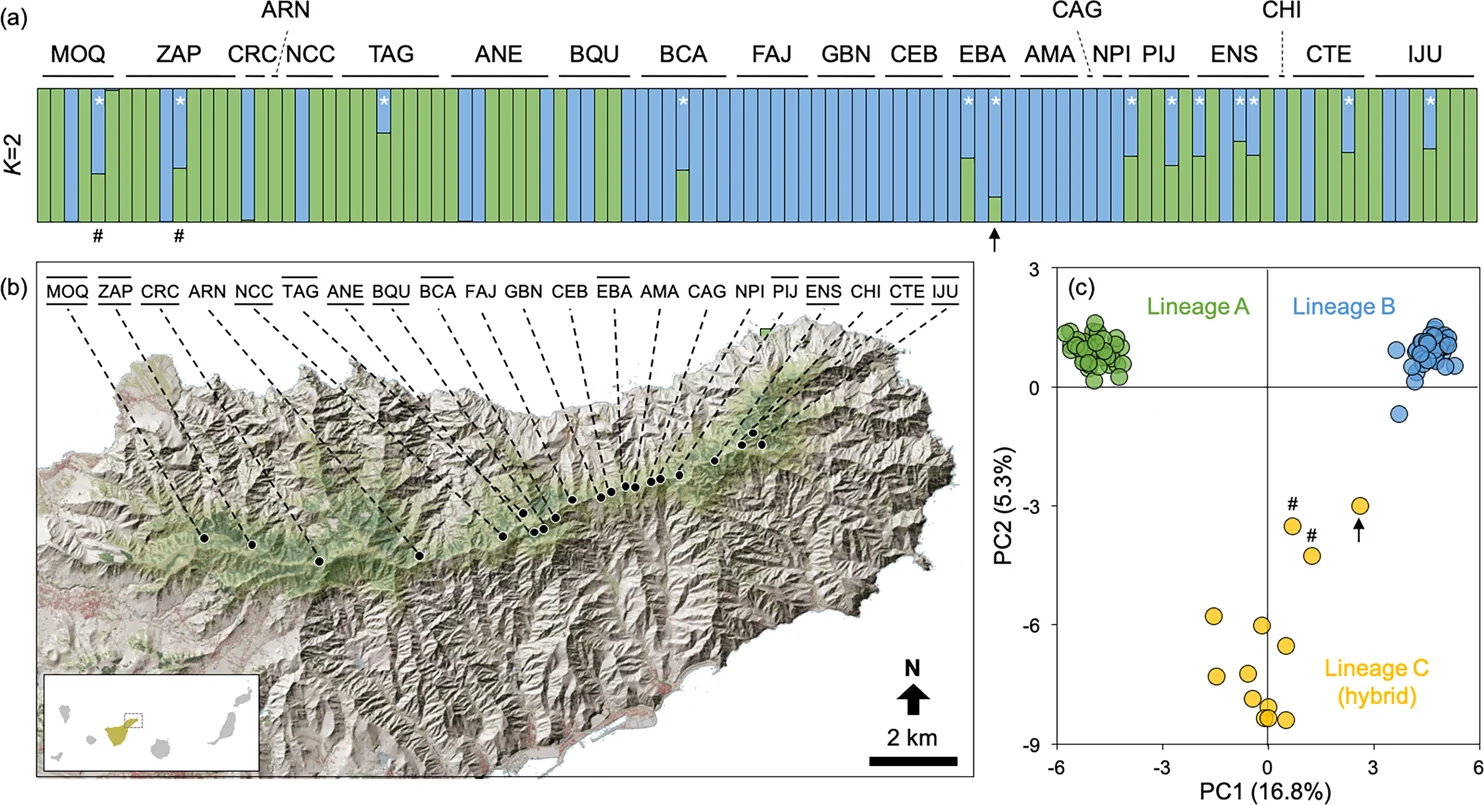
(a) Ancestry coefficients per individual within the *S. instabilis* species complex, assuming two ancestral populations (*K* = 2). (b) Geographical location of each sampling site along the Anaga peninsula. (c) Principal component analysis (PCA) of genetic variation for the sampled individuals. In the upper plot, vertical bars with a white asterisk represent individuals of admixed co-ancestry, whose position within the PCA is represented by orange circles. Black arrows in panels (a) and (c) are used to highlight the *F*_1_ between lineages B and C, while hash symbols identify the two westernmost hybrid individuals. In panel (b), underlined sampling site codes represent those where single-ancestry individuals from lineages A and B co-occur. Site codes with a line above indicate where hybrid individuals co-occur with at least one parental lineage.

### (b) Genetic relatedness using mitochondrial DNA data and *de novo* assembly of double-digestion restriction-site associated DNA sequencing data

The barcode region of the mitochondrial DNA (mtDNA) cytochrome *c* oxidase subunit I (COI) was amplified and sequenced [22]. Phylogenetic relationships among individuals were assessed with a maximum-likelihood approach as implemented in IQ-Tree 1.6.12 [23]. To generate genome-wide nuclear data, genomic DNA was processed using the double-digestion restriction-site associated DNA sequencing protocol (ddRADseq) [20,24]. Raw sequences were demultiplexed, quality-filtered and *de novo* assembled using Ipyrad 0.9.96 [25]. The supplementary material, methods S2–S4 provide details on the generation of mtDNA and genome-wide nuclear data, mtDNA-based phylogenetic analyses and ddRADseq sequence assembly, data filtering and the number of retained loci in Ipyrad (supplementary material, table S2 and figure S2).

### (c) Genomic clustering analyses

Nuclear relatedness among individuals and signatures of admixture were analysed using the Bayesian clustering method implemented in Structure 2.3.3 [26]. A hierarchical approach was used to identify the underlying genomic variation at different evolutionary scales [27,28]. Accordingly, (i) a global analysis was first conducted to evaluate overall population genetic structure across all individuals, and then (ii) analyses were repeated within inferred genetic groups to test for more fine-scale genetic structure within each.

In addition to Structure, major axes of genomic variation were visualized with a principal component analysis (PCA) using the ‘*gl.pcoa*’ function in the package *dartR* 2.9.7 [29] and R 4.2.2 [30]. Details on genomic clustering analyses are provided in the supplementary material, methods S5. Finally, pairwise-*F*_ST_ estimates and private alleles (PA) were quantified as detailed in the supplementary material, methods S5.

### (d) Analysis of nuclear genomic diversity and admixture

Elevated heterozygosity is expected in hybrid individuals as a result of the admixture of parental forms with divergent genetic backgrounds and thus carrying different alleles for a given nucleotide site [31]. Elevated heterozygosity will be highest in first-generation hybrid (*F*_1_) individuals and will diminish over time as a function of crossing dynamics and genetic drift [32]. Genome-wide nuclear data were used to estimate the observed heterozygosity (*H*_O_) per individual as implemented with the ‘*gl.report.heterozygosity*’ function of the R package *dartR*.

The presence of different hybrid classes (i.e. *F*_1_, *F*_2_, backcrosses and later-generation hybrids) was evaluated through estimating hybrid index (*Hb*_i_) and interclass heterozygosity (_i_*He*) per individual based on ancestry-informative markers (AIMs), as calculated with the R package *triangulaR* 0.0.1 [33] using an allele frequency threshold of *δ* = 0.8.

### (e) Phylogenomic inference

Evolutionary relationships were assessed through two complementary approaches using genome-wide nuclear information. First, phylogenetic relationships were reconstructed among all individuals using a maximum-likelihood approach as implemented in Raxml 8.2.12 [34]. Second, phylogenetic relationships among higher-order genetic groups detected with Structure and PCA were reconstructed via the Bayesian multispecies coalescent method implemented in Snapp 1.5.2 [35]. Details on phylogenomic inference and individual grouping for Snapp is provided in the supplementary material, methods S6.

### (f) Testing for introgression

Four-taxon ABBA-BABA tests based on *D*-statistics [36] were used to determine the role of gene flow between non-sister taxa in explaining genomic admixture patterns. Using the topology retrieved in Raxml and Snapp, where the sister lineages B and C have diverged from lineage A (supplementary material, figures S3 and S4), significantly positive or negative *D*-statistics would support the existence of gene flow between the non-sister lineages A and C or between A and B, respectively. Details on introgression tests and individual grouping are provided in the supplementary material, methods S7.

### (g) Testing alternative demographic models

A coalescent simulation-based approach was applied to statistically evaluate the fit of the observed data to alternative scenarios of divergence and hybridization (supplementary material, figure S5), as implemented in Fastsimcoal 2.5.2.21 [37]. Six competing demographic scenarios were evaluated assuming: (i) strict isolation with hierarchical divergence among lineages (‘strict bifurcation model’); (ii) directional introgression among non-sister lineages on a strict bifurcation tree (‘introgression model’); (iii) strict isolation after a single ancestral origin of all lineages (‘polytomy model’); (iv) directional introgression from one of the two parental lineages to the third putative hybrid lineage on a polytomous tree (‘polytomy followed by introgression model’); (v) the same as (iv), but for the other parental lineage; and finally (vi) fusion of the two parental lineages giving rise to a third putative hybrid lineage (‘hybrid origin model’). Details on demographic modelling are described in the supplementary material, methods S8.

### (h) Individual-based simulations of the evolution of hybrid populations

Given that the hybrid origin model was best supported by demographic inference (see results below), a custom simulation framework was used to understand how rates of drift and migration might have shaped the genomic variation of the hybrid population, in terms of the ancestry (hybrid index, *Hb*_i_) and genetic diversity (interclass heterozygosity, _i_*He*) across individuals. The potential effects of genetic linkage and incomplete RI on the trajectory of the hybrid population were also explored. Details on individual-based simulations are described in the supplementary material, methods S9.

## 3. Results

### (a) Mitochondrial data and phylogenetic relatedness

Maximum uncorrected genetic divergence between *S. instabilis* and *S. nubilosus* was 2.5%, with a maximum divergence of 1.8% within *S. instabilis*. Accordingly, IQ-Tree revealed the existence of two well-supported mtDNA monophyletic clades, corresponding to *S. instabilis* and *S. nubilosus* (supplementary material, figure S6)*.* Haplotypes within *S. instabilis* segregated into two major clades, which did not reflect any clear phylogeographic structure or an association with the genetic groups inferred using genome-wide nuclear data.

### (b) Genomic clustering analyses

Global analysis across all individuals in Structure identified the most likely number of ancestral populations to be *K* = 2, under the Δ*K* criterion (supplementary material, figure S7a). Individuals showing high single ancestry for one of the two ancestral populations were more commonly sampled in the western and eastern margins of the Anaga peninsula (hereafter ‘lineage A’), with individuals assigned to the other lineage being more commonly sampled more centrally within the peninsula (hereafter ‘lineage B’), between sampling sites BCA and NPI (figure 1a,b). However, single-ancestry individuals from both lineages were found sympatrically in nine out of the 21 sampling sites. In addition, 13 individuals with strong signatures of genetic admixture (hereafter ‘lineage C’) were sampled at nine sites, together with individuals of high single ancestry to either one or both ancestral populations (figure 1a,b). These admixed individuals showed mostly even co-ancestry coefficients (0.33 < *q*-value < 0.67), with the exception of one individual from sampling site EBA, which exhibited higher ancestry from lineage B (*q*-value = 0.81; figure 1a,c). In addition to the Δ*K* criterion, the log probability of data steadily increased until reaching an asymptote at *K* = 3, indicating that genomic variation is hierarchically structured (supplementary material, figure S7a). Clustering with *K* = 3 grouped admixed individuals into a third ancestral population of mostly single ancestry (supplementary material, figure S3), in line with global PCA inferences (figure 1c). Individuals showing an intermediate position between the two more divergent groups along the first component corresponded to those individuals showing an admixed genetic background (lineage C) for *K* = 2 in Structure (figure 1a). Genetic differentiation was higher between lineages A and B (*F*_ST-AB_ = 0.223), whereas genetic differentiation between each of these and lineage C were slightly lower and similar (*F*_ST-AC_ = 0.188; *F*_ST-BC_ = 0.190). The number of PA was higher within lineages A and B (PA_A_ = 1982, PA_AFIXED_ = 2; PA_B_ = 999, PA_B-FIXED_ = 2) than within lineage C (PA_C_ = 208, PA_C-FIXED_ = 1).

Structure analyses restricted to either lineage A or B revealed genomic variation to be structured into two geographically coherent ancestral populations within each, according to the Δ*K* criterion (supplementary material, figure S7b,c). Within each lineage, individuals of single ancestry were sampled exclusively in either westernmost or easternmost sites. Within each lineage, admixed individuals were spatially intermediate between eastern and western populations (supplementary material, figures S8 and S9). Admixed individuals within lineage A were broadly distributed in central Anaga, between the sites of ARN and BQU, while lineage B exhibited a more abrupt change of co-ancestry, which coincided with the unique sampling site of FAJ. In agreement with Structure inferences, lineage-specific PCAs described genomic variation within lineages A and B as largely organized into west and east genetic groups within each (supplementary material, figures S8 and S9).

The log probability of the data for the within-lineage Structure analyses increased beyond *K* = 2 and stabilized at *K* = 3 (supplementary material, figure S7b,c). When considering *K* = 3 in lineage A, individuals of admixed ancestry from the central sites of ARN to BQU clustered into a third ancestral population of mostly single ancestry (supplementary material, figure S8). Within lineage B, *K* = 3 defined further geographical structuring within eastern Anaga, with individuals from the easternmost sampling sites of ENS, CHI, CTE and IJU assigning with high ancestry to a third ancestral population (supplementary material, figure S9).

### (c) Analysis of nuclear genomic diversity and admixture

Levels of observed heterozygosity (*H*_O_) across individuals ranged from 0.029 to 0.074 (supplementary material, figure S3), with lineage C (admixed ancestry) presenting significantly lower *H*_O_ than that of lineages A and B (supplementary material, figure S10). According to hybrid identification analyses with *triangulaR* (figure 2), individuals within lineage C exhibited hybrid indices (*Hb*_i_) ranging from 0.38 to 0.70, with interclass heterozygosities (_i_*He*) as low as those observed within lineages A and B (figure 2b). This pattern did not align with expectations for early-generation hybrids [33], but rather with a much older origin of admixture. However, one individual assigned to lineage C presented a notably higher heterozygosity (_i_*He* = 0.35) compared to parentals, or other admixed individuals, and a skewed co-ancestry (*Hb*_i_ = 0.76) towards lineage B which was in line with its Structure-derived ancestry coefficient, suggesting it may represent a relatively recent hybrid between lineages B and C. Accordingly, when compared directly against putative parental lineages B and C, this individual exhibited an elevated heterozygosity (_i_*He* = 0.61) and a co-ancestry value close to 0.5 (*Hb*_i_ = 0.41; figure 2c), consistent with expectations for an *F*_1_ hybrid. A comparison involving lineages A and C as putative parentals revealed this hybrid individual to be more closely related to lineage C (*Hb*_i_ = 0.71), and with relatively low heterozygosity (_i_*He* = 0.30; figure 2d). Consequently, this individual was inferred to be a *F*_1_ hybrid between lineages B and C, and was discarded from further analyses.

**Figure 2.**
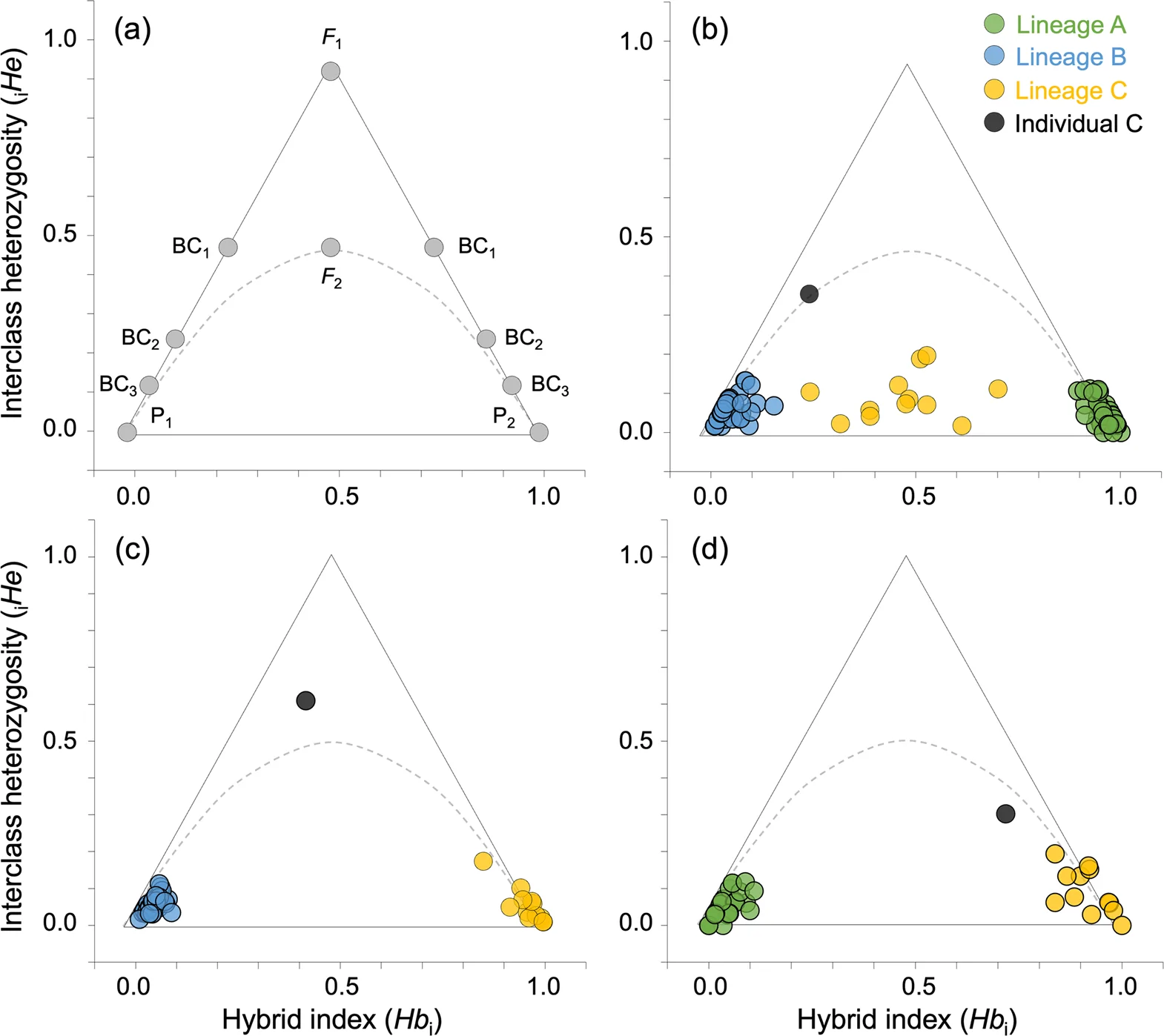
Analyses for detecting potential hybrid individuals and putative parental individuals. Panel (a) illustrates the theoretical expectations for parentals (P_i_), *F*_1_, *F*_2_ and backcrosses (BC_i_) based on their values for *Hb*_i_ and _i_*He* calculated using ancestry-informative markers (AIMs). The area under the dashed line curve would represent the position of later-generation hybrids. Panel (b) represents a test where all individuals from lineage C are compared against all individuals from lineages A and B, which are considered parentals. Panels (c) and (d) show comparisons where a given hybrid individual from lineage C (black circle) is compared against lineages B and C and lineages A and C as parentals, respectively.

### (d) Phylogenomic inference

The phylogenetic tree reconstructed in Raxml revealed three well-supported monophyletic clades (supplementary material, figure S3), consistent with the three lineages retrieved from the global Structure and PCA analyses. The Raxml topology supported a sister relationship between lineages B and C, with earlier divergence giving rise to lineage A (supplementary material, figure S3).

Coalescent reconstructions in Snapp for the three lineages derived from previous genomic clustering and accounting for geographical populations of single ancestry within lineages A and B provided similar topological inferences (supplementary material, figure S4a,b).

### (e) Introgression and alternative demographic models

ABBA-BABA tests including all individuals from lineages A and B revealed highly significant introgression between the non-sister lineages A and C (supplementary material, table S3). Significant introgression was also detected between either west or east lineages A and C. This pattern remained significant regardless of whether the west or east groups from lineages A and B were considered within the topology, suggesting an introgression event predating the geographical structuring within lineages A and B (supplementary material, table S3).

Coalescent demographic modelling in Fastsimcoal identified the ‘hybrid origin’ scenario (model vi) incorporating contemporary migration as the most-supported model (supplementary material, table S4, figure S5 and methods S8). Considering a 1-year generation time, parameter estimation within the most-supported model suggested parental lineages A and B diverged from a common ancestor approximately 0.83 Ma (*T*_DIV2_; figure 3). The origin of lineage C as a result of hybridization between parental lineages A and B was estimated to have taken place approximately 0.20 Ma (*T*_HYB_). Consistent with Structure-derived co-ancestry coefficients, the *γ* parameter estimate was 0.60 (*γ*_HYB_), suggesting a relatively even contribution of parental lineages A and B to the gene pool of lineage C (figure 1). Contemporary migration was estimated to be consistently very low across demes (figure 3).

**Figure 3.**
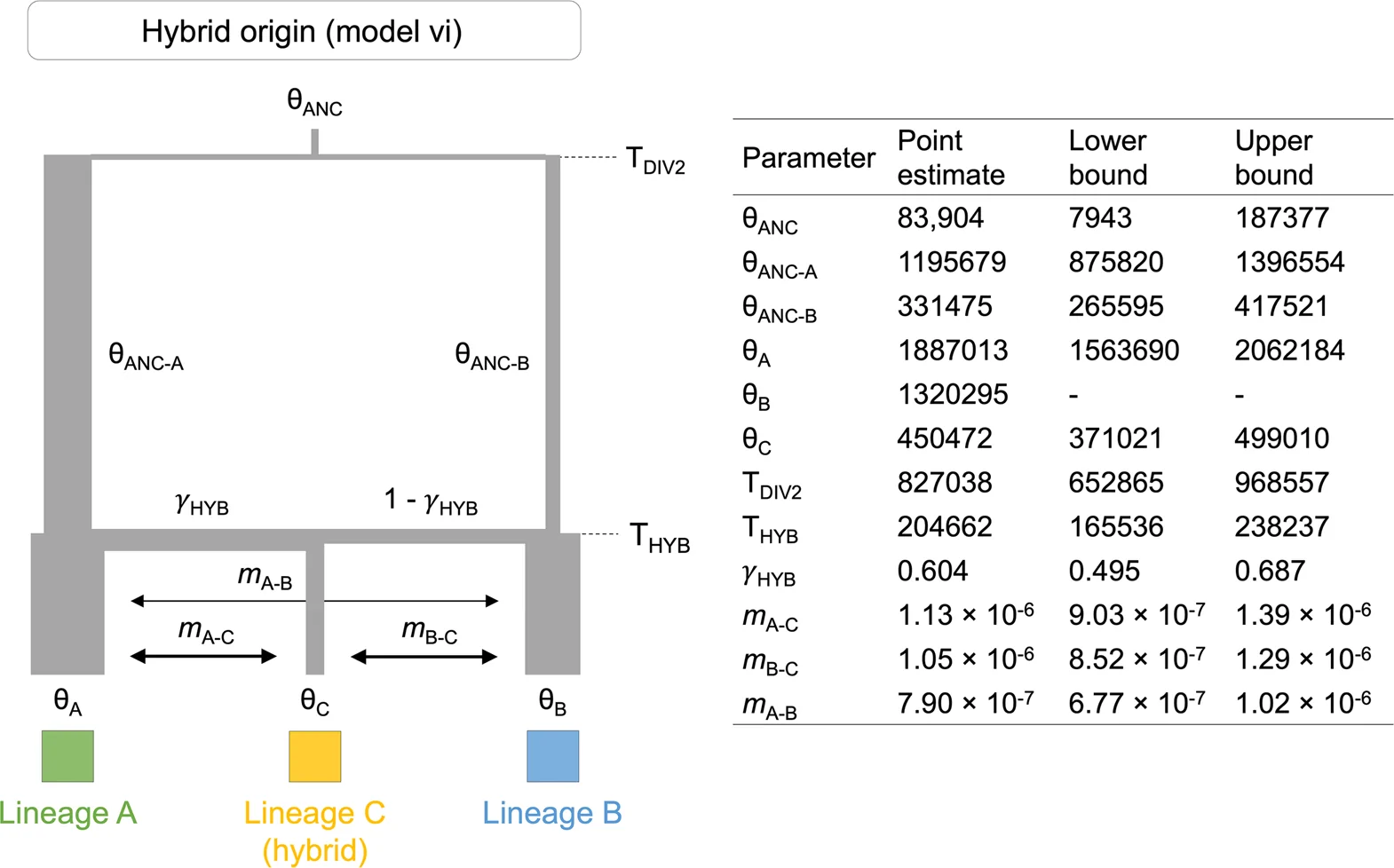
Point estimates and lower and upper 95% confidence intervals for parameters inferred with Fastsimcoal under the most-supported demographic model (model vi; supplementary material, table S4). Model parameters include ancestral (*θ*_ANC_, *θ*_ANC-A_ and *θ*_ANC-B_) and contemporary (*θ*_A_, *θ*_B_ and *θ*_C_) effective population sizes, timing of divergence (*T*_DIV2_) and hybridization (*T*_HYB_), contribution coefficient (*γ*_HYB_) and symmetrical migration rates per generation (*m*_I-J_). The relative magnitude of *θ*, *T*_DIV2_, *T*_HYB_ and *γ*_HYB_ is represented by varying width and length of branches, while arrow thickness indicates the relative magnitude of contemporary migration among demes (*m*_I-J_).

### (f) Individual-based simulations of the evolution of hybrid populations

Individual-based simulations showed that linkage relationships of ancestry informative markers could influence both the rate of loss of heterozygosity (_i_*He*) owing to drift, and the distribution of ancestry (*Hb*_i_) in hybrid populations. In simulations with free recombination (*r* = 0.5) or loose linkage, similar rates of _i_*He* loss were observed, with the hybrid population (lineage C) reaching mean levels of heterozygosity of parental lineages A and B (mean _i_*He*_AB_) at approximately 7.35 *N_e_* generations (supplementary material, figures S11a,b and S12). Although replicate simulations showed some variability in their ancestry scores, the distribution was tightly centred around 0.5 (supplementary material, figure S11c). With tight linkage (all markers clustered within 0.5 cM), the rate of _i_*He* loss was far more variable, reflecting higher stochasticity owing to the non-independence of linked sites (supplementary material, figure S12). Similarly, the limited recombination of haplotypes led to a bimodal distribution of *Hb*_i_ scores, as the populations tended to fix one of the two ancestral haplotypes (supplementary material, figures S11c and S12).

Rates of migration from parental populations also had a strong effect on the trajectory of the hybrid populations. High migration from both parental populations (*m* = 0.005) completely counteracted the loss of _i_*He* owing to drift, such that the hybrid population remained highly heterozygous even after 15 *N_e_* generations (supplementary material, figure S11d). With very low migration (*m* = 0.0001), trajectories closely resembled complete RI, but with a slower rate of loss of _i_*He.* High and intermediate levels of migration from one parent produced asymmetrical trajectories, leading the hybrid population to become swamped by the influx of parental ancestry (supplementary material, figure S11e).

These simulations indicate that the AIMs identified in the *S. instabilis* complex are loosely linked, rather than being clustered in a genomic ‘island’ of differentiation, and that the hybrid population is strongly or fully isolated from both parental lineages. Here, it is instructive to quantify the reduction in gene flow in terms of the strength of RI that would be needed to reduce mating to a given migration rate. For example, if we assume an equal probability of heterospecific (between lineage C and either lineage A or B) and homospecific encounters (within lineage C), then *m* = 0.0001 is equivalent to an estimate of RI_4_ = 0.9998 [38]. These simulations were consistent with an old hybrid population, founded approximately 7 *N_e_* generations ago (95% confidence interval: approx. 5–9 *N*_e_ generations). However, this assumes that the loss of heterozygosity was entirely owing to drift. Any selection against heterozygotes would cause heterozygosity to be purged from the hybrid population at a much faster rate than by drift alone. Thus, the above value should be considered as an upper estimate.

## 4. Discussion

Our results reveal that genetic variation for both the nuclear and mitochondrial genomes of *S. instabilis* is monophyletic with regard to *S. nubilosus*. This confirms the evolutionary independence of both taxonomic lineages and is consistent with their classification into different species. However, within *S. instabilis*, genome-wide nuclear variation clearly segregates into three genetic groups, all of which are broadly sympatric across the cloud forest of the Anaga peninsula within the island of Tenerife. The maintenance of genotype differentiation in sympatry provides strong evidence that each of the three genetic groups is a biological species. Demographic modelling with genome-wide nuclear variation provides unequivocal support for an initial cladogenetic event that underpins the origin of two species, *S. instabilis* A and *S. instabilis* B. The subsequent evolution of the third species, *S. instabilis* C, was not a cladogenetic event. Rather, it is the product of admixture involving *S. instabilis* A and B.

### (a) Hybrid speciation theory and *Silvacalles instabilis*

*Silvacalles instabilis* C is derived from hybridization between *S. instabilis* A and B, supported by coalescent demographic simulations. Additionally, the sympatric distribution of the hybrid lineage with both parental species is consistent with strong RI between *S. instabilis* C and both parental species. *Silvacalles instabilis* C thus satisfies the first two of the three conditions for a hybrid species proposed by Schumer *et al.* [5]. What remains less clear is what maintains RI and the extent to which RI may have been driven by hybridization itself, the third and controversial criterion [3] proposed by Schumer *et al.* [5] for the definition of hybrid species. However, the sympatric nature of the hybrid and parental species, together with their limited dispersal ability, and our sampling of an *F*_1_ between the hybrid species and one of the parental species, indicates an important role for post-zygotic isolation.

Our results provide evidence for different climatic optima between parental species at a broad spatial scale, but with similar or at least overlapping niche space at the local scale, providing opportunity for hybridization. Parental species sampling (figure 1; supplementary material, figure S1) suggests that *S. instabilis* A is better adapted to cooler, more humid conditions, while *S. instabilis* B is better adapted to warmer and drier conditions. Central sampling sites between BCA and NPI are drier and warmer than more western and eastern sampling sites, owing to low crestal elevation [20,39], and were exclusively sampled for *S. instabilis* B. Both *S. instabilis* A and *S. instabilis* B were sampled to the east and west of the central sites, but with *S. instabilis* A being the more abundant of the two. Given the dispersal limitation associated with flightlessness, our sampling of an *F*_1_ individual between *S. instabilis* C and *S. instabilis* B is consistent with species syntopy. The absence of later generation backcrosses argues for strong postzygotic isolation, either through hybrid sterility or hybrid breakdown.

Evidence for hybridization as a driver of speciation can be found in hybridization-derived phenotypes or incompatibilities that have acted as agents of isolation, which should not be confounded with those that are subsequent byproducts of admixture itself [6]. This criterion has focused attention on the evolution of RI in early-generation hybrids, with potential examples including *Heliconius* and *Lycaeides* butterflies [17,40,41], *Ragholetis* fruit flies [19], manakin birds [42], Darwin's finches [18] and cichlid fishes [43]. However, Long & Rieseberg [9] have highlighted the longer temporal dimension within which HHS might be expected to occur within a polygenic model. Incorporating the logic of Long & Rieseberg [9] provides for a more holistic framework for characterizing HHS, allowing for HHS to be a protracted process.

Recognized cases of HHS are typically associated with functional trait variation between parental species, where positive selection for intermediate or transgressive phenotypes simultaneously contributes to the RI of recombinant hybrid lineages [17,44,45]. Novel recombinant traits may also favour the colonization of a novel habitat by an incipient hybrid species, thus facilitating establishment and early-stage persistence by allowing it to avoid introgression and competition with the parental species [17]. Consistent with this, and based on 28 studies of homoploid hybrid species of plants, Kadereit [46] observed latitudinal/longitudinal and/or altitudinal ecogeographical displacement of hybrid lineages from parental lineages in the majority of cases. Within this framework of strong directional selection for intermediate or transgressive traits, evolution should act relatively quickly to drive speciation, analogous to a punctuated model of speciation, as has occurred with allopolyploidy diversification in plants [47]. In this context, *S. instabilis* presents an unusual case owing to its extensive sympatry among species A, B and C, their shared ecology, and the absence of obvious phenotypical or ecological differences among the three species. Given that the existence of subtle variation among species cannot be excluded, the evolution of *S. instabilis* C through positive selection for intermediate or transgressive variants cannot be discounted. However, a plausible pathway to speciation in the absence of intermediate or transgressive phenotypes is the fixation of incompatibility loci from parental species [10,12].

### (b) Selection against genetic incompatibilities and hybrid speciation

The conditions for hybrid speciation are enhanced when a hybrid population maintains a mixed combination of parental genetic incompatibilities that contribute to RI with both parental species. Theoretical work has demonstrated how reciprocal sorting of BDM genetic incompatibilities [48–50] from parental species can result in hybrid speciation, even in cases where hybrids are selected against. Using simulations, Schumer *et al.* [10] have shown that this is more likely for co-evolving or adaptive incompatibility loci, where hybrid speciation can occur even in the face of ongoing gene flow from parental species and strong selection against hybrids. Such adaptively evolving BDM incompatibility loci diverge between populations in response to positive directional or divergent selection. Thus, they are expected to evolve more rapidly and thus provide a faster pathway to hybrid speciation, compared to neutrally evolving BDM incompatibility loci, where alternative alleles are fixed within different populations by genetic drift. However, Blanckaert & Bank [12], again using simulations, have demonstrated that hybrid speciation can also occur when parental BDM loci are evolving neutrally, and that speciation becomes highly probable under certain conditions. Within their neutral BDM model, Blanckaert & Bank [12] highlight an apparent paradox. Barriers need to be weak enough to allow the formation of the hybrid population in the first place. However, at the same time, barriers need to be strong enough for the hybrid population to be isolated from both parental populations. This paradox would be resolved within a dynamic where genomic reproductive isolating barriers between parental species are weak enough to allow a hybrid population to form, but with a subsequent period of geographical isolation of the hybrid population from parental species. Genomic signatures within *S. instabilis* are consistent with a geographically localized hybrid origin, and this has occurred within a topoclimatic arena within which isolation among populations is favoured during glacial periods [20,39].

### (c) Western origin and eastward range expansion of the hybrid species

A striking feature of genomic variation within *S. instabilis* C is the extent of genotypic differentiation among individuals, compared with the more limited differentiation seen within both parental species (figures 1c and 2b). Increased genotype diversity relative to parental populations is an expected outcome within early-generation hybrid crosses and backcrosses, as recombinant genotypes emerge from divergent parental allelic variation. However, this explanation can be discounted within *S. instabilis* C, as individuals are not early-generation hybrid crosses and backcrosses, as evidenced by the absence of elevated heterozygosity relative to the parental species (figure 2b). In contrast, a consideration of geographical patterns of individual relatedness both within and among species provides some explanation for the origin and significance of genotypic diversity within *S. instabilis* C.

Together with the *F*_1_ individual between *S. instabilis* B and C, the two westernmost individuals of *S. instabilis* C (sampled from MOQ and ZAP, figure 1b) present displacement in PCA space toward *S. instabilis* B (figure 1c). The relatedness patterns for these two individuals may be explained by two competing scenarios. Within scenario one, subsequent to the origin of *S. instabilis* C, geographically localized introgression within the west of Anaga may have occurred from *S. instabilis* B to C. Within scenario two, the western extreme of Anaga may represent the geographical origin of *S. instabilis* C within Anaga*,* with subsequent range expansion to the east. Within this second scenario, higher sharing of ancestral variation would be expected between western populations of *S. instabilis* C and both parental species, with drift effects leading to increasing levels of differentiation of newly founded populations of *S. instabilis* C and both parental species, as range expansion of *S. instabilis* C occurs to the east. Both scenarios make testable predictions for geographical patterns of relatedness both within and among species.

Scenario one predicts higher relatedness of western individuals of *S. instabilis* C to *S. instabilis* B, but not to *S. instabilis* A. By contrast, scenario two predicts higher relatedness of western individuals of *S. instabilis* C to both parental species. Support for scenario two is provided by PCA analyses where only one of the parental species is included together with *S. instabilis* C (supplementary material, figure S13). In both PCAs, both western individuals of *S. instabilis* C are displaced towards the parental species. In both cases, they are specifically displaced towards parental individuals from western sampling sites, consistent with an origin of admixture in the west. Further to this, in both PCAs, *S. instabilis* C presents a geographical gradient of displacement from parental variation, with higher displacement associated with the more eastern sites of ENS, CTE and IJU. Results are thus consistent with a geographically localized western hybrid origin for *S. instabilis* C. Understanding how this western admixed population could have remained geographically isolated for some period from more eastern populations of the parental species requires consideration of how Quaternary climate oscillations have driven population isolation and secondary contact within dispersal-limited insect species of the Anaga cloud forest [20,39].

### (d) Isolation of hybrid population from parental species

Cloud forest connectivity and fragmentation within the Anaga peninsula have been moderated by climate oscillations throughout the Quaternary. Extensive evidence for this dynamic is provided by isolation and admixture dynamics within multiple beetle species, including *S. instabilis* [20,22,39,51]. Genetic variation within cloud forest beetle species is frequently structured into west and east populations, highlighting a dominant topoclimatic feature that has promoted cycles of population isolation and secondary contact during the Quaternary [20]. Contemporary patterns of gene flow across the area of secondary contact highlight how admixture can emerge locally [20].

Within the broader regional genetic structure of species identified by Salces-Castellano *et al.* [20], finer-scale geographical structuring of genetic variation within regions is not uncommon [22,39,51], and is indeed apparent within *S. instabilis* B, which presents strong population substructure within the very east of its range (supplementary material, figure S9). Such intra-regional structure is probably driven by past or present environmental barriers to gene flow related to hydric stress landscapes [20]. With regard to the western origin and inferred isolation of the hybrid species, several species of beetle provide evidence for past barriers to dispersal that are geographically coincident with this inference. *Geomitopsis franzi* (Staphylinidae) presents east–west structuring consistent with that observed by Salces-Castellano *et al.* [20], with further structure to the west [52]. This western structure involves three deeply divergent mtDNA lineages, with secondary contact between two of them occurring approximately 1000 m west–southwest of site MOQ (figure 1b). Genome-wide nuclear genetic variation in the beetle species *Tarphius canariensis* (Zopheridae) also reveals strong geographical structuring of genetic variation between site MOQ and further sampling 1000 m west–southwest of MOQ [53].

*Geomitopsis franzi* and *T. canariensis* highlight a dynamic of geographical isolation and secondary contact within the western limits of the Anaga cloud forest. This is geographically coincident with where nuclear genomic data for *S. instabilis* predicts the origin of *S. instabilis* C, thus providing a mechanistic escape from the apparent paradox of Blanckaert & Bank [12]. Admixture of parental populations and a resulting hybrid swarm in the western limits of the Anaga cloud forest, with coincident or subsequent isolation from eastern populations of the parental species during glacial conditions, would provide time for reciprocal sorting of genetic incompatibilities of parental species. Inferences from both demographic modelling and individual-based simulations are consistent with a hybrid origin over such a time scale. Higher cloud forest connectivity during interglacial warming [39] would facilitate subsequent sympatry among all three species through westward range expansion of parental species and eastward range expansion of the hybrid species. It is also plausible that admixture and isolation of *S. instabilis* C occurred further west of the Anaga peninsula, either driven directly by the dramatic flank collapses and volcanic activity across the north of the island [54] or indirectly through topoclimatic phenomena [51].

Implicit within this scenario is the local breakdown of RI between parental species *S. instabilis* A and B in the western limits of Anaga, while RI is maintained between more eastern populations. This implies geographical variation in hybridization dynamics, something that has been observed across different hybrid zones between hermit and Townsend's warblers (*Dendroica occidentalis* and *Dendroica townsendi*) [55] and varieties of the common snapdragon (*Antirrhinum majus* subspecies *majus* variety *pseudomajus* and *Antirrhinum majus* subspecies *majus* variety *striata*) [56]. In the case of *S. instabilis*, a potential explanation for geographical variation in historical gene flow between parental species A and B is provided by the species pair, *Tarphius canariensis* and *T. simplex*, both co-distributed with *S. instabilis*. Higher historical gene flow between western sympatric populations of the two *Tarphius* species, relative to eastern sympatric populations, coincides with a west–east gradient of increasing genomic divergence between populations of both species, resulting from east-west range expansions across the peninsula for both [22]. Thus, similar to both species of *Tarphius*, increased RI between *S. instabilis* A and B may have been facilitated by the emergence of higher genomic divergence through range expansion dynamics.

### (e) Hybridization as a facilitator of speciation?

Our data provide strong evidence for a hybrid origin for *S. instabilis* C, and for its status as a biological species. However, determining a causative role for hybridization in the evolution of RI between *S. instabilis* C and its parental species is elusive, and highlights a more general challenge to understand the extent to which hybridization may play a role in speciation. Hybrid speciation theory suggests that HHS times may range from tens of generations [10] to many thousands of generations [12], depending upon the evolutionary characteristics of genomic incompatibilities, linkage architecture and recombination rates. Accepted cases of HHS are associated with the evolution of RI over relatively short time scales [5], where hybridization is likely to have been a driving agent of speciation through direct selection for hybrid phenotypes [17,18,42], or background selection against genomic incompatibilities arising from adaptively evolving or coevolving loci [10]. Evolution of RI over longer timescales, as might be expected when BDM incompatibilities have evolved neutrally, is suggested to be potentially more common than has been thought [9]. However, while the evolution of RI involving BDM incompatibilities that have evolved neutrally is theoretically possible, it requires strong isolation of hybrid populations from parental species [12]. The evolution of *S. instabilis* C could be explained within such a dynamic through a period of geographical isolation of the hybrid population from the parental species.

Hybridization can thus be considered not only a fast-acting driver for speciation (i.e. selection acting on hybrid phenotypes or adaptively evolving BDM loci) but also a slower-acting facilitator of speciation (i.e. selection acting on neutrally evolving BDM loci). This recognizes the relationship between hybridization and speciation as a spectrum, ranging from a primary driver, through to a role of facilitation, or to no appreciable role in the establishment of RI. While such a framework does not lessen the challenges to address and quantify the role of hybridization in the evolution of RI, it does embrace the concerns stemming from the limitations that overly strict criteria may impose on the understanding of general principles [3]. By considering hybridization as a facilitator of speciation, one can ask what conditions might favour the formation of hybrid populations in the first place, a necessary prerequisite to facilitation. In the case of *S. instabilis* C, it is noteworthy that both parental range sizes have been strongly impacted by Quaternary climate oscillations, and that dispersal ability is sufficiently limited to maintain genetic isolation among allopatric populations. If speciation is facilitated by the formation of hybrid populations, then time to speciation (i.e. RI = 1) for such populations from their parental species should be less than that of the parental species through cladogenesis. Our coalescent simulations are consistent with this. Despite hybridization between parental species being approximately four times more recent than cladogenetic speciation between parental species, RI of the hybrid species already approaches that observed between parental species. While this circumstantial evidence is consistent with hybrid speciation, it remains speculative until direct evidence for the role of BDM loci can be obtained. Here, we note that the unique characteristics of the *S. instabilis* system provide a framework for testing such a role.

A detailed exploration of locus-level ancestry patterns within lineage C (supplementary material, figure S14) reveals parental allelic variation to be differently fixed across the range of lineage C, for which two divergent and non-exclusive hypotheses can be invoked. The first is that speciation occurred quickly within an allopatric hybrid population when it formed in the west, such that much of the ancestral variation was still present within the hybrid lineage at the time of range expansion to the east, with geographically independent sorting of this variation across the expanded range. The second hypothesis is of a longer period of isolation of the hybrid population in the west, within which ancestral variation sorted to expectations for population equilibrium. Within such a scenario, inter-individual variance for ancestral variation would have been more limited across the range of *S. instabilis* C after expansion to the east, and observed variance would be explained by subsequent local introgression from parental species. Within both scenarios, if selection acted against allelic combinations of low fitness across BDM loci, this should yield signatures of low genetic variation within *S. instabilis* C that correspond to genomic regions of low genetic variation within, but high genomic differentiation between the parental species *S. instabilis* A and *S. instabilis* B.

## 5. Conclusions

*Silvacalles instabilis* provides strong evidence for RI between a species of hybrid origin and both parental species. The parental species *S. instabilis* A and *S. instabilis* B are estimated to have diverged approximately 800 000 years ago, with subsequent hybridization giving rise to *S. instabilis* C approximately 200 000 years ago. An ancient rather than recent hybrid origin for *S. instabilis* C is also supported by genome-wide nuclear estimates of heterozygosity and individual-based simulations of hybrid origin. Based on geographical patterns of genomic relatedness between hybrid and parental individuals, the most plausible explanation is that *S. instabilis* C evolved within a hybrid swarm that remained geographically isolated from both parental species long enough for RI to emerge. The phenotypic, ecological and temporal dimensions of speciation within *S. instabilis* suggest the need for a more nuanced consideration of the role of hybridization in speciation. Much focus has been placed on hybridization as a driving agent of speciation. We argue that hybridization may also play a facilitative role. This recognizes the speciation relevance of hybridization within a spectrum, ranging from a primary driver, through a gradient of facilitation, to the other extreme of no appreciable role in the establishment of RI. We suggest that isolated hybrid swarm populations are likely to evolve RI faster than allopatric isolates of parental populations, and that this may be a useful framework to understand hybridization as a facilitator of speciation.

## Supplementary Material



## Data Availability

Illumina reads are deposited at the NCBI Sequence Read Archive (SRA) under BioProject PRJNA1356498, with BioSample accessions SAMN53060348-SAMN53060457. DNA sequences for the COI gene fragment are deposited in GenBank with accession numbers PX489569-PX489673. Input files for all analyses are available for download from the Figshare data repository [57]. The individual-based simulation code used in this study is available on Zenodo [58]. All supplementary tables, figures and methods cited in the main text have been uploaded online as supplementary material [59].
