# Supplementary material for: Hybrid species evolution between cryptic and sympatric weevil species
Source: Proc Biol Sci. 2026 Oct 28;293(2081):20261745. doi: 10.1098/rspb.2026.1745 (PMC13725538; doi:10.1098/rspb.2026.1745)

Proceedings of the Royal Society B: Biological Sciences

Electronic Supplementary Material (ESM)

DOI: https://doi.org/10.1098/rspb.2026.1745

Hybrid species evolution between cryptic and sympatric weevil species

Víctor Noguerales[1,2*], Marius H. Eisele[1], Sophia L. Gray[3], Sean Stankowski[4] & Brent C. Emerson[1*]

[1]Institute of Natural Products and Agrobiology (IPNA-CSIC), San Cristóbal de La Laguna, Canary Islands, Spain.

[2]Instituto de Investigación en Recursos Cinegéticos (IREC - UCLM, CSIC, JCCM), Ciudad Real, Spain.

[3]Department of Ecology and Evolution, University of Sussex, Brighton BN1 9RH, UK.

[4]Department of Genetics, Evolution, and Environment, University College London. London UK, WC1E 6BT.

***Authors for correspondence:**

Víctor Noguerales: *victor.noguerales@csic.es*

Brent C. Emerson: *bemerson@ipna.csic.es*

SUPPLEMENTARY INFORMATION

SUPPLEMENTARY METHODS

Methods S1. Sample collection

Specimens from the *Silvacalles instabilis* (Wollaston, 1864) species complex were collected within the cloud forest of the Anaga peninsula, located on the Canary island of Tenerife (Figure 1). To augment the ten sampling sites included originally in Salces-Castellano *et al*. (2020), additionally sampling was conducted giving rise to a total of 110 specimens from a total of 21 sampling sites (Figure 1; Table S1), including 106 individuals from *S. instabilis*, and 4 individuals from the sister species *S. nubilosus*, which were included as an outgroup in downstream genomic analyses when required. The geographic distance between sampling sites ranged from 0.2 km to 14 km along the dorsal ridge of the Anaga peninsula, with a maximum elevational difference between sites of 200 m (Figure S1; Table S1). Sampling was performed following the protocol described in Salces-Castellano *et al*. (2020) and Emerson *et al*. (2017). Briefly, target species were sampled by beating overhead vegetation onto a light-coloured umbrella. Then, fallen individuals were collected from the umbrella, preserved in 100% ethanol and stored at -20°C till DNA extraction. All sampled individuals came from overhead vegetation, including shrubs, tree branches and dead or decaying trunks. Many of the sampled individuals were collected within the course of a broader sampling campaign to address community-level questions using the beetle assemblage from the cloud forest of the Anaga peninsula (*e.g*., Salces-Castellano *et al*., 2020), for which host plants were not recorded. Posterior sampling targeting the *Silvacalles instabilis* species complex also did not record host plant use. Sampling was undertaken with permits issued by 'Cabildo de Tenerife' (*Expte: AFF17/23, Nº Sigma: 2023-00133*).

Methods S2. Mitochondrial and genome-wide nuclear data

Genomic DNA was extracted from each individual using the Biosprint DNA Blood Kit (Qiagen) on a Thermo KingFisher Flex automated extraction instrument. The barcode region of the mitochondrial DNA (mtDNA) cytochrome c oxidase subunit I (COI) was amplified for each individual using the primers *Fol-degen-for* and *Fol-degen-rev* (Yu *et al*., 2012), following conditions described in Noguerales and Emerson (2025). PCR products were purified with enzymes *ExoI* and *rSAP* (New England Biolabs, Ipswich, MA, USA), and then Sanger sequenced (Macrogen, Madrid, Spain), edited with GENEIOUS PRIME version 2021.1.1 and aligned using MAFFT (FFT-NS-i method; Katoh & Standley, 2013).

To generate genome-wide nuclear data, individual genomic DNA was processed using the double-digestion restriction-site associated DNA sequencing protocol (ddRADseq, Peterson *et al*., 2012), as described in Salces-Castellano *et al*. (2020), and Noguerales and Emerson (2025). Individual DNA extracts were digested with the restriction enzymes *MseI* and *EcoRI* (New England Biolabs, Ipswich, MA, USA). Resulting genomic libraries were then pooled at equimolar ratios, followed by size selection for fragments between 200 and 300 base pairs (bp), and paired-end sequenced (150 bp) on an Illumina NovaSeq6000 (Novogene, Cambridge, UK).

Methods S3. Genetic relatedness using mtDNA data

Phylogenetic relationships among all individual mtDNA sequences were assessed with a maximum-likelihood approach as implemented in IQ-TREE version 1.6.12 (Trifinopoulos *et al.*, 2016). A total of 1000 ultrafast bootstrapping replicates were conducted for node support calculation (Minh *et al.*, 2013), implementing an HKY+I model of nucleotide evolution according to the Bayesian information Criterion (BIC), as calculated by MODELFINDER within IQ-TREE (Kalyaanamoorthy *et al.*, 2017). Four individuals belonging to the sister species *Silvacalles nubilosus* were used as an outgroup.

Methods S4. Genomic data filtering and sequence assembly

First, the overall quality of raw Illumina reads was visually inspected using FASTQC version 0.11.7 (Andrews, 2010). Raw reads were then demultiplexed, quality filtered and *de novo* assembled using IPYRAD version 0.9.96 (Eaton & Overcast, 2020). A stricter filter to remove Illumina adapter contamination (*filter_adapters*) was applied and only reads with unambiguous barcodes (*max_barcode_mismatch*) were retained. After trimming restriction overhangs for enzymes *EcoR1* and *MseI* (*restriction_overhang*), base calls with a Phred score <20 were converted into ambiguous sites (Ns) and reads with >5 Ns (*max_low_qual_bases*) were discarded. Afterwards, the retained reads within and across samples were clustered considering a threshold of sequence similarity of 85% (*clust_threshold*), and those clusters with a minimum coverage depth of less than 5 (*mindepth_majrule*) and a maximum coverage depth of more than 10,000 (*maxdepth*) were discarded. Statistical base calling was performed at a minimum depth of 6 (*mindepth_statistical*). Resulting loci shorter than 35 base pairs (bp) (*filter_min_trim_len*) and with more than 20% polymorphic sites (*max_SNPs_locus*) were discarded. The maximum proportion of shared heterozygous sites in a locus (*max_shared_Hs_locus*) was set to 50%. In a final filtering step, only loci that were present in at least 80% of the samples (*min_samples_locus*) were retained.

A total of 241.17 million raw sequences across the 110 individuals were retrieved: 106 individuals of *Silvacalles instabilis* and 4 specimens belonging to the sister species *S. nubilosus* (Figure S2). After the filtering steps in IPYRAD, each individual retained an average of 2.19 million reads (SD=1.85) (Figure S2a) and 40,671 clusters (SD=14,371) (Figure S2b) of high-quality sequences, with a mean depth per locus of 33.01. The on average length of the retained loci was 92.1 bp (SD=20.8 bp), which contained on average 9.3 segregating sites (SD=5.0). This yielded a total of 5040 unlinked SNPs when including all 106 specimens, 5250 unlinked SNPs when including only the 45 individuals from lineage A, 7927 unlinked SNPs when including only the 48 individuals from lineage B, and 7022 unlinked SNPs when including only individuals of hybrid origin (13 specimens from the admixed lineage C). On average, missing data in each SNP matrix was approximately 10.88%-13.64%. According to IPYRAD, estimates of sequencing error rates and heterozygosity across the 106 individuals were on average 0.00103 (SD=0.00033) and 0.01785 (SD=0.00325), respectively. Unless otherwise indicated (see phylogenetic analyses in RAXML), all downstream analyses were performed using datasets of unlinked SNPs (*i.e.*, a single SNP per RAD

locus). A summary of the number of individuals and loci included in each of the subsequent analyses is provided in Tables S2-S3.

Methods S5. Genomic clustering analyses

Nuclear relatedness among individuals and the potential signatures of admixture between them were examined using the Bayesian Markov Chain Monte Carlo (MCMC) clustering method implemented in STRUCTURE version 2.3.3 (Pritchard *et al.,* 2000). STRUCTURE was run with 200,000 MCMC cycles after a burn-in step of 100,000 iterations, assuming correlated allele frequencies and admixture (Pritchard *et al.,* 2000) and performing 10 independent runs for each value of *K* ancestral populations (from *K*=1 to *K*=5). The most likely number of ancestral populations was estimated after retaining the 10 runs per each *K*-value with the highest likelihood estimates. Convergence across runs was assessed by checking that the 10 retained replicates per *K*-value provided a similar solution in terms of individual probabilities of assignment to a given ancestral population (*q*-values; Gilbert *et al.,* 2012). As recommended by Gilbert *et al.* (2012) and Janes *et al.* (2017), two statistics were used to interpret the number range of ancestral populations (*K*) that best describes our data: log probabilities of Pr(X|*K*) (Pritchard *et al.,* 2000) and Δ*K* (Evanno *et al.,* 2005), both calculated in STRUCTURE HARVESTER (Earl & vonHoldt, 2012). Finally, the Greedy algorithm in CLUMPP version 1.1.2 was used to align replicated runs of STRUCTURE for the same *K*-value (Jakobsson & Rosenberg, 2007). In addition to STRUCTURE, major axes of genomic variation were visualised with a Principal Component Analysis (PCA) using the '*gl.pcoa'* function in the package *dartR* 2.9.7 (Mijangos *et al.*, 2022) and R 4.2.2 (R Core Team, 2022). As for STRUCTURE, a hierarchical approach was followed, thus PCAs were conducted at different evolutionary scales, both across all individuals and within each of the resulting genetic groups. Following Ortego and Noguerales (2025), both STRUCTURE and Principal Component Analysis (PCA) were run using datasets only including SNPs with a minor allele frequency (MAF) >3%, using the '*gl.filter.maf*' function as implemented in the R package *dartR* version 2.9.7 (Mijangos *et al.*, 2022) and R version 4.2.2 (R Core Team, 2022). Finally, to measure genetic differentiation between genetic groups and detect diagnostic alleles within each, pairwise-F_{ST} estimates and private alleles (PA) were quantified using the '*gl.fst.pop*' and '*gl.report.pa*' functions, respectively, within the R package *dartR*.

Methods S6. Phylogenomic inferences

Phylogenetic relationships among all individuals were reconstructed using a maximum-likelihood (ML) approach as implemented in RAXML version 8.2.12 (Stamatakis, 2014). This analysis was performed on a matrix of concatenated SNPs (*i.e.*, including all SNPs per RAD locus), which was edited with the R package *phrynomics* (B. Banbury, *http://github.com/bbanbury/phrynomics*) to remove invariant and non-binary SNPs, resulting in a dataset of 20,407 concatenated SNPs. The ascertainment bias correction based on the conditional likelihood method (Lewis, 2001) and the GTR-GAMMA model of nucleotide evolution were applied. The best-scoring maximum likelihood tree and node supports were estimated through 100 rapid bootstrap replicates. The 4 individuals from the sister species *Silvacalles nubilosus* (Table S1) were used as an outgroup.

Phylogenetic relationships among higher-order genetic groups were reconstructed using SNAPP version 1.5.2 (Bryant *et al.*, 2012), a coalescent-based method for species tree estimation, as implemented in BEAST version 2.6.7 (Bouckaert *et al.*, 2014). Phylogenetic inference in SNAPP was conducted using two individual grouping strategies: (i) individuals were assigned to the main genetic groups identified within the global analyses of both STRUCTURE and PCA, and; (ii) to take into account geographical structuring of genetic variation within the higher-order genetic groups, individuals were assigned into geographic populations of single co-ancestry, based on results of within-lineage analyses by both STRUCTURE and PCA. Accordingly, first individuals were assigned to each of the three genetic groups (either lineage A, B or C), according to the clustering scheme observed in the global PCA and STRUCTURE inferences, assuming *K*=2 (Figure 1, Figure S3). To reduce the high computational burden of SNAPP, a total of 20 individuals for each of the two main genetic groups (lineages A and B) were selected, encompassing the overall within-lineage genetic variation observed in their respective clustering analyses (Figures S8-S9). Regarding lineage C, the individual classified as an F_1 hybrid with lineage B was excluded (Figure 1ac), yielding a total of 12 individuals of admixed co-ancestry. The final dataset contained 52 individuals distributed across the three demes. Second, phylogenetic relationships using SNAPP were also reconstructed by taking into account the geographical structuring of genetic variation within each of the two main lineages (lineages A and B). For each lineage, individuals were assigned to geographically coherent populations according to the clustering schemes observed in the within-lineage PCA and STRUCTURE analyses, assuming *K*=2 (Figures S8-S9). Accordingly, a total of 12 individuals of high single ancestry (*q*-value >0.95) were selected for each of the genetic groups, corresponding to western and eastern lineage A, and western and eastern lineage B. Regarding lineage C, the aforementioned subset of 12 individuals was included. The final dataset contained 60 individuals distributed across the five demes.

For each of the two datasets, the respective *.usnps* file from IPYRAD was edited with the R package *phrynomics* (B. Banbury, *https://github.com/bbanbury/phrynomics*) to remove non-binary SNPs, which resulted in matrices including 5615 and 5199 bi-allelic unlinked SNPs shared across demes, respectively. The default values for the shape (α) and inverse scale (β) parameters of the gamma prior distribution for the population size parameter (θ) were set, and the forward (*u*) and reverse (*v*) mutation rates were left to be calculated by SNAPP. The log-likelihood correction was applied, and the coalescent rate was set to be sampled, with the remaining parameters left as default. Two independent runs using different starting seeds for 1 million MCMC generations were performed, sampling every 1000 steps (~1000 genealogies). The log files were examined in TRACER version 1.7 (Rambaut *et al.*, 2018) to check stationarity and convergence of the chains and confirm that effective sample sizes (ESS) for all parameters were >200. The software LOGCOMBINER version 2.4.7 (Drummond & Rambaut, 2007) was used to remove 10% of trees as burn-in and combine tree and log files for replicated runs. Finally, TREEANNOTATOR version 2.4.7 (Drummond & Rambaut, 2007) and DENSITREE version 2.2.6 (Bouckaert, 2010) were used to obtain the maximum credibility trees and displayed the full set of retained trees, respectively.

Methods S7. Introgression analyses using ABBA-BABA tests

Potential signatures of gene flow between non-sister taxa were examined using four-taxon ABBA-BABA tests based on the *D*-statistics (Durand *et al.*, 2011), as implemented in IPYRAD (Eaton &

Overcast, 2020). This method enables the evaluation of the extent to which an excess of shared alleles in a given lineage is the result of either historical gene flow from a non-sister lineage, or retention of ancestral genetic variation (*i.e.*, incomplete lineage sorting, ILS). Standard deviations of *D*-statistics were obtained through 1000 bootstrap replicates. The two grouping strategies used for the phylogenomic analyses in SNAPP were also employed for ABBA-BABA tests. Assuming the topology retrieved in RAXML (Figures S3), firstly all individuals were assigned to one of the three lineages A, B and C, according to the clustering pattern observed in the global PCA and STRUCTURE analyses. Then, ABBA-BABA tests were run using the same subset of 52 individuals used in SNAPP for reconstructing the phylogenetic relations among these three higher-order genetic groups (Figures S4a). Second, structuring of genetic variation within the higher-order genetic groups was taken into account. Accordingly, individuals were grouped into geographic populations of single co-ancestry on the basis of the clustering schemes observed in the within-lineage analyses and *D*-statistics were recalculated. Then, ABBA-BABA tests were run using the same subset of 60 individuals used in SNAPP to take into account the geographical structuring of genetic variation within each of the two main lineages (lineages A and B) (Figures S4b). For all analyses, the 4 individuals of *Silvacalles nubilosus* were used as an outgroup.

Methods S8. Testing alternative models of divergence and hybridisation in FASTSIMCOAL

To evaluate the relative statistical support for each of the alternative demographic scenarios (Figure S5, Table S4), the composite likelihood of the observed data given a specified model was estimated in FASTSIMCOAL version 2.5.2.21 (Excoffier *et al.*, 2021) using the site frequency spectrum (SFS). Six competing demographic scenarios were evaluated assuming: (i) strict isolation with hierarchical divergence among lineages ('strict bifurcation model'); (ii) directional introgression among non-sister lineages on a strict bifurcation tree ('introgression model'); (iii) strict isolation after a single ancestral origin of all lineages ('polytomy model'); (iv) directional introgression from one of the two parental lineages to the third putative hybrid lineage on a polytomous tree ('polytomy followed by introgression model'); (v) the same as (iv), but for the other parental lineage, and finally; (vi) fusion of the two parental lineages giving rise to a third putative hybrid lineage ('hybrid origin model'). These six models were evaluated either assuming contemporary symmetrical migration among all demes, or the absence of migration, thus yielding a total of twelve competing demographic scenarios (Figure S5). These analyses were conducted using the same subset of 52 individuals selected for reconstructing phylogenetic relationships in SNAPP among the higher-order genetic groups. For demographic models i and ii, the topology inferred from phylogenomic analyses in RAXML and SNAPP was used. The folded joint SFS was calculated using EASYSFS version 0.0.1 (I. Overcast, *https://github.com/isaacovercast/easySFS*), considering a single SNP per locus to avoid the effects of linkage disequilibrium. Each of the three genetic groups was downsampled to ~80% of individuals to remove all missing data for the calculation of the SFS, minimise errors with allele frequency estimates, and maximise the number of variable SNPs retained. The final SFS contained 4272 variable SNPs. Because invariable sites were not included in the SFS, the *'removeZeroSFS'* option in FASTSIMCOAL was applied. Accordingly, the effective population size (N_e) for one of the demes (lineage A) was fixed to enable the estimation of other parameters (Papadopoulou & Knowles, 2015; Excoffier *et al.*, 2021; Noguerales & Ortego, 2022). To this end, the parameter N_e was calculated from observed estimates of

nucleotide diversity (π) and mutation rate per site per generation (μ), where N_e=π/4μ. The software DNASP version 6.12.03 (Rozas *et al.,* 2017) was used to estimate π for the fixed deme using phased data from polymorphic and non-polymorphic loci contained in the *.allele* file from IPYRAD. A mutation rate per site per generation of 2.8×10^{-9} (Keightley *et al.*, 2014), as estimated for the butterfly *Heliconius melpomene*, was considered.

A total of 100 independent replicated runs were conducted for each model, considering 100,000-250,000 simulations for the calculation of the composite likelihood, 10-40 expectation-conditional maximisation (ECM) cycles, and a stopping criterion of 0.001 (Excoffier *et al.*, 2021). An information-theoretic model selection approach based on Akaike's information criterion (AIC) was applied to determine the probability of each model given the observed data (Burnham & Anderson, 2002; *e.g.*, Thomé & Carstens, 2016). The replicated run for each model with the highest composite likelihood was selected to calculate its respective AIC score, as detailed in Thomé and Carstens (2016). Then, AIC values were rescaled across models, calculating the difference between the AIC value of a given model and the minimum AIC obtained among all competing models (*i.e.*, the best model has ΔAIC=0). The point estimates of the different demographic parameters for the most-supported model were selected from the run with the highest maximum composite likelihood. Finally, the confidence intervals of the parameter estimates (based on the percentile method; *e.g.*, de Manuel *et al.*, 2016) were estimated under the most-supported model through 100 parametric bootstrap replicates by simulating SFS from the maximum composite likelihood estimates and re-estimating parameters each time (Excoffier *et al*., 2021). Coalescent demographic modelling in FASTSIMCOAL identified the 'hybrid origin' scenario (model vi) incorporating contemporary migration as the most-supported model (Table S4; Figure S5). Support for the next best model, the 'introgression model' (model ii), was substantially less with ΔAIC > 20. Among the remaining models, the scenario assuming a strict bifurcation divergence was the least supported (Table S4). The subset of models assuming no contemporary migration were much more poorly supported than their homologous scenarios considering contemporary migration (ΔAIC>480; Table S4, Figure S5).

Methods S9. Individual-based simulations of the evolution of hybrid populations

Given that the hybrid origin model was best supported by demographic inference (see Results section), a custom simulation framework (S. L. Gray, *https://github.com/sophiagray2001/hybridsimulator.git*; also available on Zenodo, *https://doi.org/10.5281/zenodo.21645107*) was used to understand how rates of drift and migration might have shaped the genomic variation of the hybrid population, in terms of the ancestry (hybrid index, Hb_i) and genetic diversity (interclass heterozygosity, $_iHe$) across individuals. The basic model consisted of two ancestral populations, each consisting of 100 individuals, that carry a number of ancestry-informative markers (AIMs) with allele frequencies and rates of missing data that match the empirical dataset for *triangulaR* analysis (*i.e.*, loci with δ≥0.8). These populations mate to form a population of 200 F_1 hybrids. The hybrid population is then allowed to evolve for 3000 generations (equivalent to 15N_e generations), with each mating pair leaving 2 offspring. As for the detection of different hybrid classes (*i.e.*, F_1, F_2, backcrosses and later-generation hybrids) using the R package *triangulaR* 0.0.1 [33], individuals were assigned

to putative parental and hybrid populations according to the main genetic groups inferred with clustering analyses (STRUCTURE and PCA).

A total of 13 variations on this basic model (50 independent replicates each) were explored. First, because the genomic distribution of loci is currently unknown, how the degree of linkage affects the trajectory of the hybrid populations was explored through simulating the joint distribution of Hb_i and $_iHe$ under five alternative scenarios. These scenarios assumed: (i) free recombination among loci; (ii) the random assignment of loci to 20 linkage groups with rates of recombination between neighboring loci defined by their map distance (each replicate with a different randomly generated map); (iii) all markers assigned to a single chromosome; (iv) tight linkage where all loci were clustered within 0.5 centimorgans (cM) of one another, and; (v) clustering with no recombination. For simulations with linkage, genetic markers were randomly arrayed onto *n* chromosomes, each with dimensions 0,1. To simulate free recombination, each marker was assigned to separate chromosomes. For the scenarios with 20 chromosomes and 1 chromosome, each chromosome pair experienced 1 crossover per generation. For the tight linkage scenario, the per-chromosome recombination rate was reduced to 0.05, and for the scenario with no recombination no crossovers were allowed to occur.

Following this, eight additional scenarios were built to explore the effect of incomplete RI. These scenarios allowed either gene flow from one parental population (scenarios vi-ix) or both parental populations (scenarios x-xiii) into the hybrid population, with each assuming four rates of migration (m=0.005, m=0.001, m=0.0005 and m=0.0001). To allow for low rates or migration, parental individuals were introduced in the population as rate *m*. Because the population size is relatively small, low migration was simulated by only introducing migrants at specific intervals. For example, to achieve an overall rate of m=0.001, a parental migrant was introduced every 5 generations.

References

Andrews S. 2010. FASTQC: A quality control tool for high throughput sequence data. *http://www.bioinformatics.babraham.ac.uk/projects/fastqc/*

Bouckaert R, Heled J, Kuehnert D, Vaughan T, Wu C-H, Xie D, Suchard MA, Rambaut A, Drummond AJ. 2014. BEAST2: A software platform for Bayesian evolutionary analysis. *PLOS Computational Biology* 10: e1003537.

Bouckaert RR. 2010. DENSITREE: Making sense of sets of phylogenetic trees. *Bioinformatics* 26: 1372-1373.

Bryant D, Bouckaert R, Felsenstein J, Rosenberg NA, RoyChoudhury A. 2012. Inferring species trees directly from biallelic genetic markers: Bypassing gene trees in a full coalescent analysis. *Molecular Biology and Evolution* 29: 1917-1932.

Burnham KP, Anderson DR. 2002. *Model selection and multimodel inference. A practical information-theoretic approach*. 2nd ed. Springer-Verlag, New York.

de Manuel M, Kuhlwilm M, Frandsen P, Sousa VC, Desai T, Prado-Martinez J, Hernandez-Rodriguez J, Dupanloup I, Lao O, Hallast P, Schmidt JM, Heredia-Genestar JM, Benazzo A, Barbujani G, Peter BM, Kuderna LFK, Casals F, Angedakin S, Arandjelovic M, Boesch C, Kuehl H, Vigilant L, Langergraber K, Novembre J, Gut M, Gut I, Navarro A, Carlsen F, Andres AM., Siegismund HR, Scally A, Excoffier L, Tyler-Smith C, Castellano S, Xue Y, Hvilsom C, Marques-

Bonet T. 2016. Chimpanzee genomic diversity reveals ancient admixture with bonobos. *Science* 354: 477-481.

Durand EY, Patterson N, Reich D, Slatkin M. 2011. Testing for ancient admixture between closely related populations. *Molecular Biology and Evolution* 28: 2239-52.

Drummond AJ, Rambaut A. 2007. BEAST: Bayesian evolutionary analysis by sampling trees. *BMC Evolutionary Biology* 7: 214.

Earl DA, vonHoldt BM. 2012. STRUCTURE HARVESTER: A website and program for visualizing STRUCTURE output and implementing the Evanno method. *Conservation Genetics Resources* 4: 359-361.

Eaton DAR, Overcast I. 2020. IPYRAD: Interactive assembly and analysis of RADseq datasets. *Bioinformatics* 36: 2592-2594.

Emerson BC, Casquet J, Lopez H, Cardoso P, Borges PAV, Mollaret N, Oromí P, Strasberg D, Thebaud C. 2017. A combined field survey and molecular identification protocol for comparing forest arthropod biodiversity across spatial scales. *Molecular Ecology Resources* 17: 694-707.

Evanno G, Regnaut S, Goudet J. 2005. Detecting the number of clusters of individuals using the software STRUCTURE: A simulation study. *Molecular Ecology* 14: 2611-2620.

Excoffier L, Marchi N, Marques DA, Matthey-Doret R, Gouy A, Sousa VC, Schwartz R. 2021. FASTSIMCOAL2: Demographic inference under complex evolutionary scenarios. *Bioinformatics* 37: 4882-4885.

Gilbert KJ, Andrew RL, Bock DG, Franklin MT, Kane NC, Moore J-S, Moyers BT, Renaut S, Rennison DJ, Veen T, Vines TH. 2012. Recommendations for utilizing and reporting population genetic analyses: The reproducibility of genetic clustering using the program STRUCTURE. *Molecular Ecology* 21: 4925-4930.

Jakobsson M, Rosenberg NA. 2007. CLUMPP: A cluster matching and permutation program for dealing with label switching and multimodality in analysis of population structure. *Bioinformatics* 23: 1801-1806.

Janes JK, Miller JM, Dupuis JR, Malenfant RM, Gorrell JC, Cullingham CI, Andrew RL. 2017. The *K*=2 conundrum. *Molecular Ecology* 26: 3594-3602.

Kalyaanamoorthy S, Minh BQ, Wong TKF, von Haeseler A, Jermiin LS. 2017. MODELFINDER: Fast model selection for accurate phylogenetic estimates. *Nature Methods* 14: 587-589.

Katoh K, Standley DM. 2013. MAFFT - Multiple sequence alignment software version 7: improvements in performance and usability. *Molecular Biology and Evolution* 30: 772-780.

Keightley PD, Ness RW, Halligan DL, Haddrill PR. 2014. Estimation of the spontaneous mutation rate per nucleotide site in a *Drosophila melanogaster* full-sib family. *Genetics* 196: 313-320.

Lewis PO. 2001. A likelihood approach to estimating phylogeny from discrete morphological character data. *Systematic Biology* 50: 913-925.

Mijangos JL, Gruber B, Berry O, Pacioni C, Georges A. 2022. *dartR* v2: An accessible genetic analysis platform for conservation, ecology and agriculture. *Methods in Ecology and Evolution* 13: 2150-2158.

Minh BQ, Nguyen MAT, von Haeseler A. 2013. Ultrafast approximation for phylogenetic bootstrap. *Molecular Biology and Evolution* 30: 1188-1195.

Noguerales V, Ortego J. 2022. Genomic evidence of speciation by fusion in a recent radiation of grasshoppers. *Evolution* 76: 2618-2633.

Noguerales V, Emerson BC. 2025. Arthropod mtDNA paraphyly: A case study of introgressive origin. *Journal of Evolutionary Biology* 38: 272-283.

Ortego J, Noguerales V. 2025. Mountain speciation driven by high rates of lineage formation and rapid evolution of partial reproductive isolation: insights from a recent radiation of grasshoppers (Orthoptera: Gomphocerinae). *Zoological Journal of the Linnean Society* 205: zlaf141.

Papadopoulou A, Knowles LL. 2015. Genomic tests of the species-pump hypothesis: Recent island connectivity cycles drive population divergence but not speciation in Caribbean crickets across the Virgin Islands. *Evolution* 69: 1501-1517.

Peterson BK, Weber JN, Kay EH, Fisher HS, Hoekstra HE. 2012. Double digest RADseq: An inexpensive method for *de novo* SNP discovery and genotyping in model and non-model species. *Plos One* 7: e37135.

Pritchard JK, Stephens M, Donnelly P. 2000. Inference of population structure using multilocus genotype data. *Genetics* 155: 945-959.

Rambaut A, Drummond AJ, Xie D, Baele G, Suchard MA. 2018. Posterior summarization in Bayesian phylogenetics using TRACER 1.7. *Systematic Biology* 67: 901-904.

R Core Team. 2022. R: A language and environment for statistical computing. R Foundation for Statistical Computing, Vienna. Available at https://www.r-project.org/.

Rozas J, Ferrer-Mata A, Sánchez-del Barrio JC, Guirao-Rico S, Librado P, Ramos-Onsins SE, Sánchez-Gracia A. 2017. DNASP 6: DNA sequence polymorphism analysis of large data sets. *Molecular Biology and Evolution* 34: 3299-3302.

Salces-Castellano A, Patiño J, Alvarez N, Andújar C, Arribas P, Braojos-Ruiz JJ, del Arco-Aguilar M, García-Olivares V, Karger DN, López H, Manolopoulou I, Oromí P, Pérez-Delgado AJ, Peterman WE, Rijsdijk KF, Emerson BC. 2020. Climate drives community-wide divergence within species over a limited spatial scale: evidence from an oceanic island. *Ecology Letters* 23: 305-315.

Stamatakis A. 2014. RAXML version 8: A tool for phylogenetic analysis and post-analysis of large phylogenies. *Bioinformatics* 30: 1312-1313.

Thomé MTC, Carstens BC. 2016. Phylogeographic model selection leads to insight into the evolutionary history of four-eyed frogs. *Proceedings of the National Academy of Sciences of the United States of America* 113: 8010-8017.

Trifinopoulos J, Nguyen LT, von Haeseler A, Minh BQ. 2016. W-IQ-TREE: A fast online phylogenetic tool for maximum likelihood analysis. *Nucleic Acids Research 44*: W232-W235.

Yu DW, Ji Y, Emerson BC, Wang X, Ye C, Yang C, Ding Z. 2012. Biodiversity soup: Metabarcoding of arthropods for rapid biodiversity assessment and biomonitoring. *Methods in Ecology and Evolution* 3: 613-623.

SUPPLEMENTARY TABLES

Table S1. Geographic coordinates and elevation for each of the sampling sites (Figure S1). For each site, the total number of individuals from the *Silvacalles instabilis* species complex with mitochondrial (mtDNA) and genome-wide nuclear (nDNA) data is indicated. Four individuals belonging to the sister species *Silvacalles nubilosus* were used as an outgroup, for which mtDNA and nDNA data was also generated.

Site name	Site code	Longitude	Latitude	Elevation (meters)	*N* total (mtDNA)	*N* total (nDNA)	*N* outgroup (mtDNA/nDNA)
MOQ	T21	-16.308815	28.536802	778	6	6	-
ZAP	T20	-16.296557	28.535704	883	9	9	-
CRC	T19	-16.280031	28.532019	931	2	2	-
ARN	T18	-16.249380	28.533854	744	1	1	-
NCC	T17	-16.232687	28.538624	831	4	4	-
TAG	T16	-16.226058	28.544338	808	8	8	-
ANE	T15	-16.225111	28.540000	886	7	8	-
BQU	T14	-16.222923	28.541069	774	6	6	2
BCA	T13	-16.219854	28.542549	739	7	7	1
FAJ	T12	-16.215988	28.546845	701	6	6	-
GBN	T11	-16.209503	28.547270	713	5	5	-
CEB	T10	-16.207486	28.548379	681	5	5	-
EBA	T9	-16.203886	28.549813	675	5	5	-
AMA	T8	-16.201273	28.549903	716	5	5	-
CAG	T7	-16.196037	28.550428	707	1	1	-
NPI	T6	-16.194065	28.551440	705	2	2	-
PIJ	T5	-16.189222	28.551972	791	4	5	-
ENS	T4	-16.179648	28.555401	801	5	6	-
CHI	T3	-16.173594	28.559029	879	1	1	1
CTE	T2	-16.171389	28.562028	805	5	6	-
IJU	T1	-16.169194	28.560194	755	7	8	-

Table S2. Number of individuals and loci included in each of the analyses. All analyses were performed using datasets of unlinked SNPs (*i.e.*, a single SNP per RAD locus), except phylogenetic analyses in RAXML which was run on a matrix of concatenated SNPs (*i.e.*, including all SNPs per RAD locus). STRUCTURE and principal component analysis (PCA) were run using datasets only including unlinked SNPs with a minor allele frequency (MAF) >3%**.** The estimation of hybrid index (Hb_i) and interclass heterozygosity ($_iHe$) for evaluating the presence of different hybrid classes was performed using ancestry-informative markers (AIMs) based on unlinked SNPs.

**Analysis**	**Subset of individuals and lineages**	***N* of individuals**	***N* of loci**
STRUCTURE + PCA	Lineages A, B and C	106	1603 unlinked SNPs
STRUCTURE + PCA	Lineage A	45	2281 unlinked SNPs
STRUCTURE + PCA	Lineage B	48	4105 unlinked SNPs
PCA	Lineages A and C	61	2082 unlinked SNPs
PCA	Lineages B and C	58	3213 unlinked SNPs
Observed heterozygosity (H_O)	Lineages A, B and C	106	5040 unlinked SNPs
Observed heterozygosity (H_O)	Lineage A	45	5250 unlinked SNPs
Observed heterozygosity (H_O)	Lineage B	48	7927 unlinked SNPs
F_{ST} + private alleles	Lineages A, B and C	106	5040 unlinked SNPs
Triangle plots for Hb_i and $_iHe$	Lineages A, B and C	106	65 AIMs
Triangle plots for Hb_i and $_iHe$	Lineages A and C	58	38 AIMs
Triangle plots for Hb_i and $_iHe$	Lineages B and C	61	134 AIMs
RAXML	Lineages A, B, C, and outgroups	110	20407 concatenated SNPs
SNAPP	Lineages A, B and C	52	5615 unlinked SNPs
SNAPP	Western and eastern lineages A and B, and lineage C	60	5199 unlinked SNPs
ABBA-BABA tests	Lineages A, B, C, and outgroups	110	4457 unlinked SNPs
FASTSIMCOAL	Lineages A, B and C	52	4761 unlinked SNPs

Table S3. Analyses of introgression using *D*-statistics (ABBA-BABA tests), assuming the topological inferences obtained in RAXML and SNAPP. The value of the *D*-statistics, its standard deviation (SD), *Z*-score and *P*-value are provided for each test. Assuming that the sister taxa P_1 and P_2 diverged from P_3, the *D*-statistic is used to test the null hypothesis of no introgression (*D*-statistics=0) between P_3 and P_1 or P_2. *D*-statistics significantly different from 0 indicate gene flow between P_2 and P_3 (*D*-statistics > 0) or between P_1 and P_3 (*D*-statistics < 0). Analyses were performed (i) using all individuals in each higher-order genetic group, corresponding to lineages A, B and C (tests 1-2), (ii) including the same subset of 20 individuals from each lineage A and B (tests 3-4), as used for SNAPP and FASTSIMCOAL (see Supplementary Methods S4-S6), and (iii) taking into account the structuring of genetic variation within the higher-order genetic groups, and therefore assigning individuals into geographic populations of single co-ancestry, according to the clustering schemes observed in the within-lineage analyses (tests 5-12). All tests were repeated after excluding the F_1 individual between lineages B and C (Figure 1, Figure 2), giving rise to a P_2 group composed of 12 individuals. For all analyses, the four individuals from *S. nubilosus* were used as an outgroup.

Test	P_1	P_2 (hybrid)	P_3	*D*-statistics	SD	*Z*-score	*P*-value
1	Lineage B (all ind.)	Lineage C (12 ind.)	Lineage A (all ind.)	0.084	0.017	5.121	<0.001
2	Lineage B (all ind.)	Lineage C (all ind.)	Lineage A (all ind.)	0.082	0.016	5.563	<0.001
3	Lineage B (20 ind.)	Lineage C (12 ind.)	Lineage A (20 ind.)	0.088	0.017	5.162	<0.001
4	Lineage B (20 ind.)	Lineage C (all ind.)	Lineage A (20 ind.)	0.085	0.017	5.063	<0.001
5	Western lineage B (12 ind.)	Lineage C (12 ind.)	Western lineage A (12 ind.)	0.073	0.018	4.088	<0.001
6	Eastern lineage B (12 ind.)	Lineage C (12 ind.)	Western lineage A (12 ind.)	0.112	0.018	6.388	<0.001
7	Western lineage B (12 ind.)	Lineage C (all ind.)	Western lineage A (12 ind.)	0.070	0.017	4.115	<0.001
8	Eastern lineage B (12 ind.)	Lineage C (all ind.)	Western lineage A (12 ind.)	0.110	0.017	6.370	<0.001
9	Western lineage B (12 ind.)	Lineage C (12 ind.)	Eastern lineage A (12 ind.)	0.110	0.018	6.118	<0.001
10	Eastern lineage B (12 ind.)	Lineage C (12 ind.)	Eastern lineage A (12 ind.)	0.074	0.019	3.966	<0.001
11	Western lineage B (12 ind.)	Lineage C (all ind.)	Eastern lineage A (12 ind.)	0.108	0.017	6.222	<0.001
12	Eastern lineage B (12 ind.)	Lineage C (all ind.)	Eastern lineage A (12 ind.)	0.072	0.018	3.879	<0.001

Table S4. Comparison of the alternative demographic scenarios of divergence and hybridisation within the *Silvacalles instabilis* species complex, tested using FASTSIMCOAL (Figure S5). The most-supported model is highlighted in bold (Figure 3). Each topological model was evaluated either assuming contemporary symmetrical migration among all demes or the lack of it, yielding a total of twelve competing demographic scenarios.

**Model**	**Contemporary migration**	***k***	**lnL**	**AIC**	**ΔAICC**	**ω_i**
(i) Strict bifurcation	No	6	-8315.54	16643.08	702,06	0.00
(i) Strict bifurcation	Yes	9	-7995,63	16009.26	68.24	0.00
(ii) Introgression	No	9	-8216,65	16451.30	510.29	0.00
(ii) Introgression	Yes	12	-7968.91	15961.82	20.80	0.00
(iii) Polytomy	No	4	-8391.93	16791.85	850.84	0.00
(iii) Polytomy	Yes	7	-8042,51	16099.02	158.00	0.00
(iv) Polytomy + introgression	No	6	-8288.81	16589.61	648.59	0.00
(iv) Polytomy + introgression	Yes	9	-7986.56	15991.11	50.10	0.00
(v) Polytomy + introgression	No	6	-8300.38	16612.75	671.73	0.00
(v) Polytomy + introgression	Yes	9	-7986.49	15990.98	49.96	0.00
(vi) Hybrid origin	No	8	-8214,11	16444,23	503.21	0.00
**(vi) Hybrid origin**	**Yes**	**11**	**-7959,51**	**15941,02**	**0.00**	**1.00**

k: number of parameters in the model; lnL: maximum likelihood estimate of the model; AIC: Akaike's information criterion value; ΔAIC: difference between a given model and the best-ranked; ω_i: AIC weight.

SUPPLEMENTARY FIGURES

Figure S1. Geographic location and elevation of each sampling site along the Anaga peninsula. For each sampled site, the occurrence of individuals belonging to the three lineages from the *Silvacalles instabilis* species complex is provided in the upper plot (Figure 1). A missing-colored circle denotes that no individual from that specific lineage was sampled in a given site.

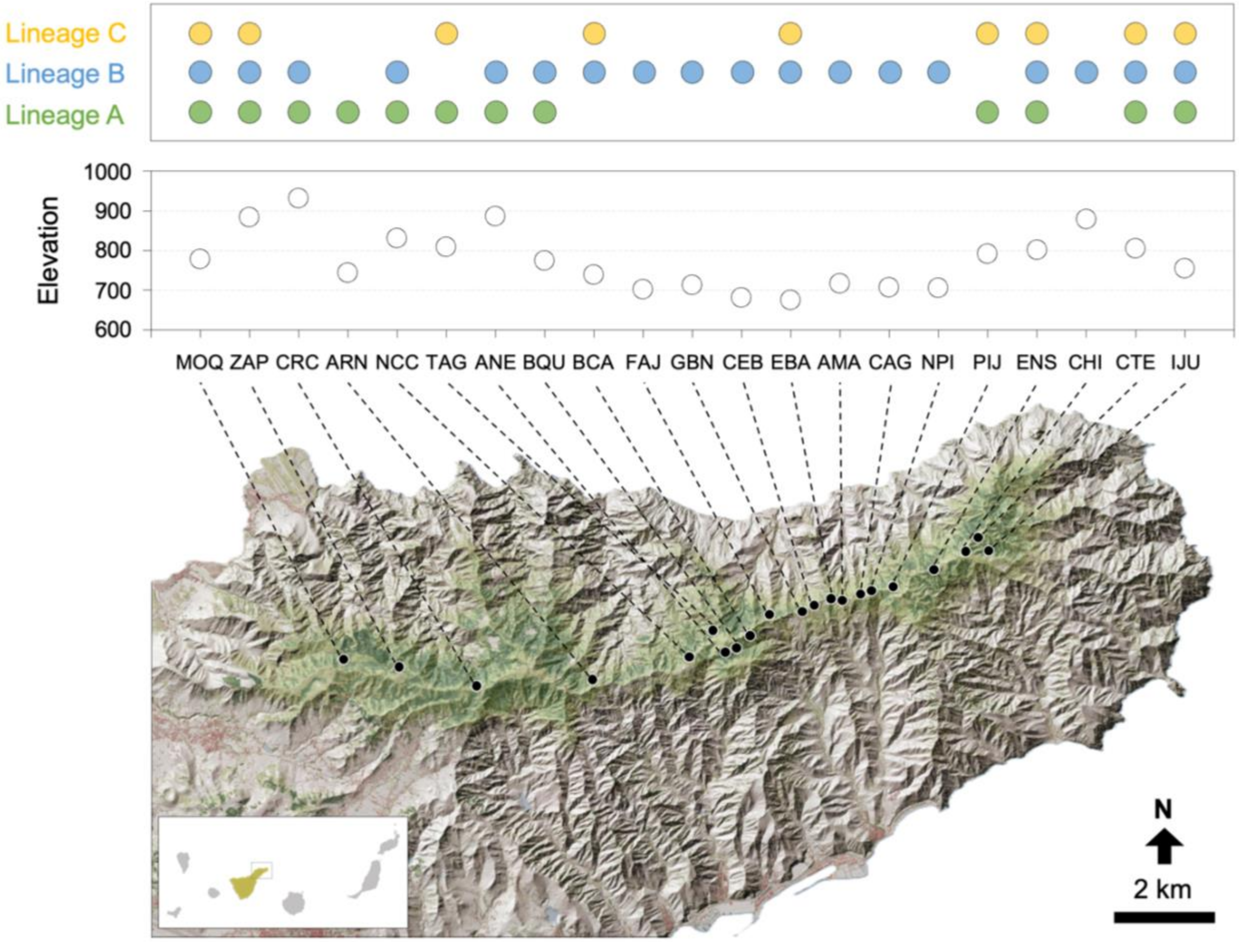

Figure S2. The upper plot shows the number of reads per individual after the different quality filtering steps conducted in IPYRAD. The cumulative stacked bars in the bottom plot summarise the number of sequence clusters before and after the assembly and filtering steps in IPYRAD. Within each bar, the pale grey colour represents the clusters that were discarded during the filtering procedure, with the dark grey colour representing the total number of retained clusters. Site codes as in Table S1.

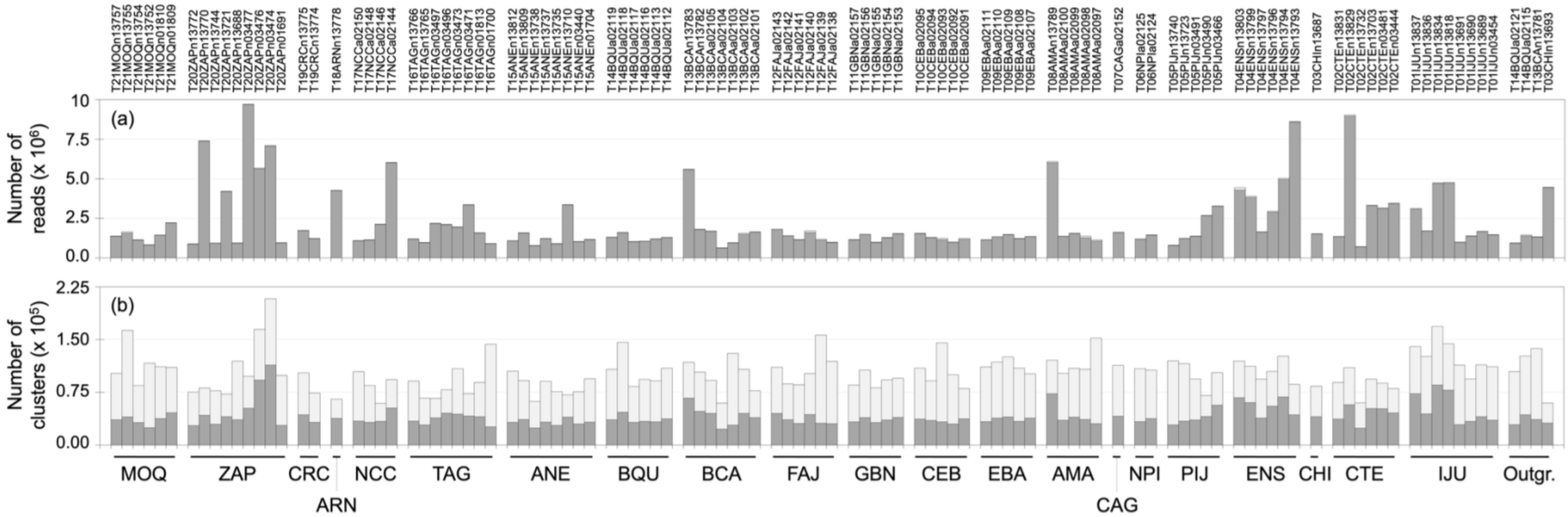

Figure S3. Phylogenetic relationships among individuals of the *Silvacalles instabilis* species complex, as inferred from genome-wide nuclear data using RAXML. Asterisks on the tree denote fully supported nodes (bootstrap value >99). Colour barplots represent the individual-based ancestry coefficients as inferred in STRUCTURE, assuming two and three ancestral populations (K=2-3). For each individual, the right grey bar represents its genetic variability as measured using the observed heterozygosity (H_O) parameter. Four individuals belonging to the sister species *S. nubilosus* were used as an outgroup. Sites codes are as in Table S1, with colours as in Figure 1.

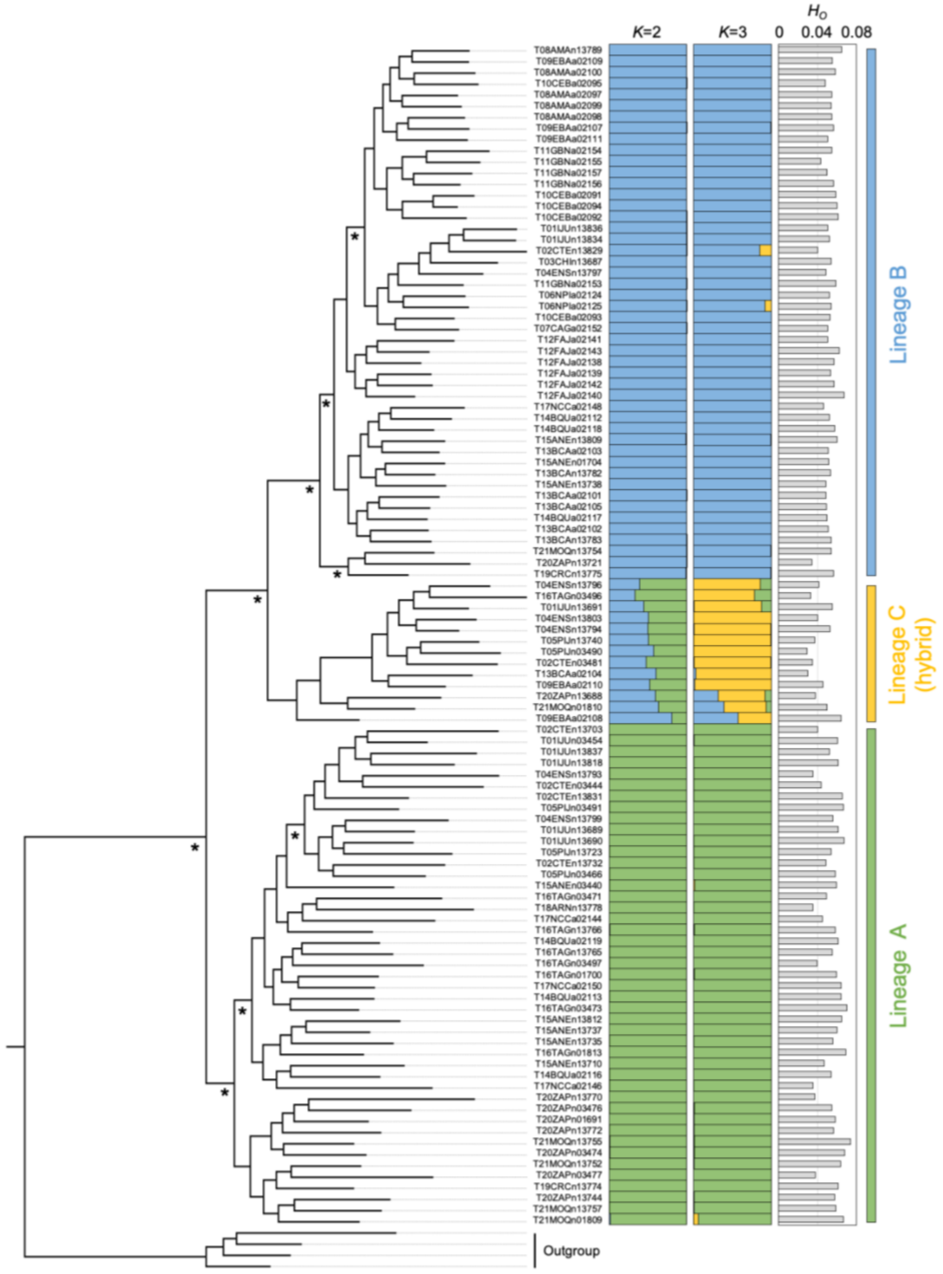

Figure S4. Phylogenetic relationships as inferred in SNAPP among (a) the three higher-order genetic groups within the *Silvacalles instabilis* species complex, and (b) their geographic populations according to the clustering schemes observed in the within-lineage analyses by STRUCTURE. All nodes are fully supported.

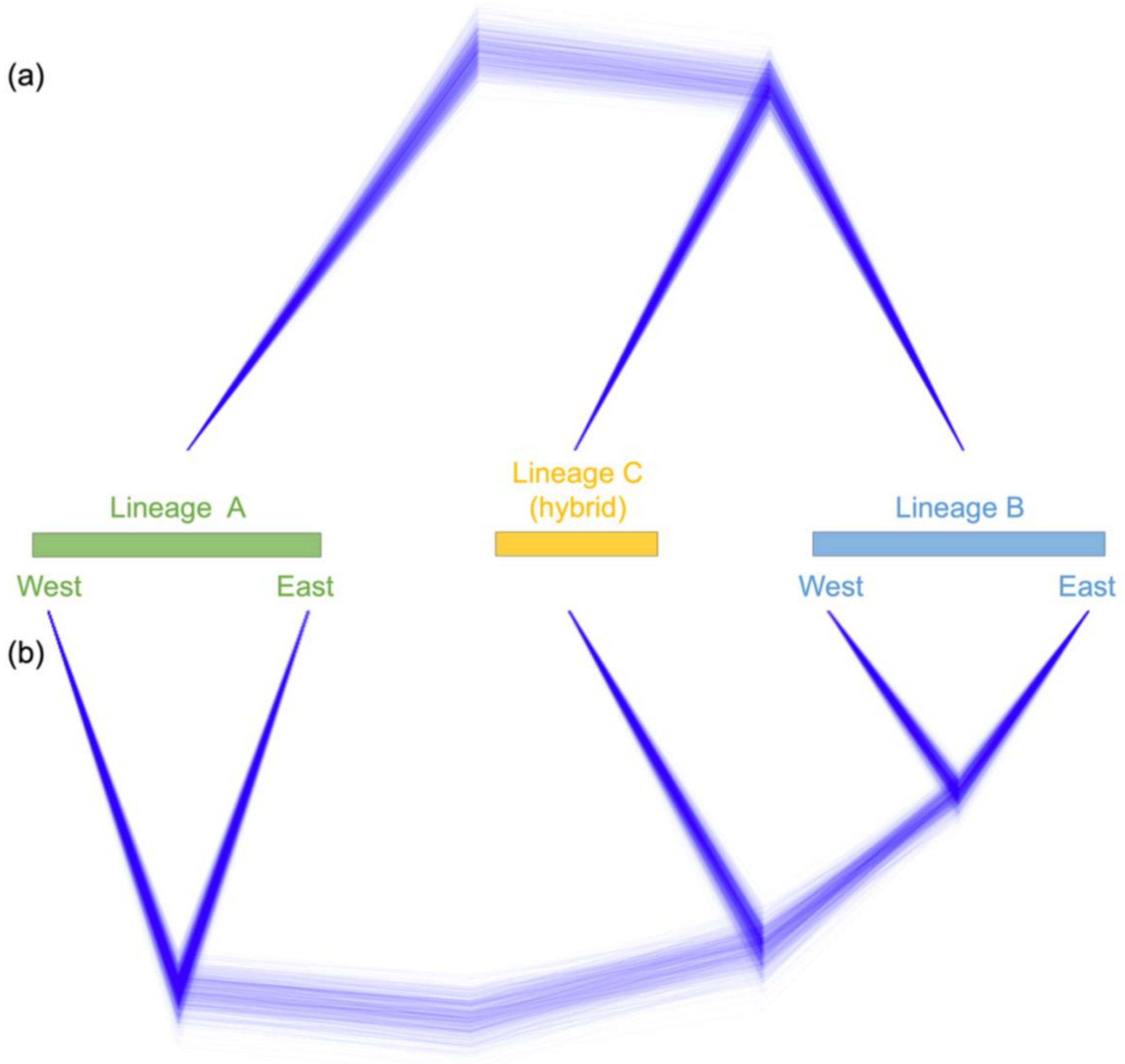

Figure S5. Alternative demographic scenarios of divergence and hybridisation within the *Silvacalles instabilis* species complex, tested using FASTSIMCOAL. Model parameters include ancestral (θ_{ANC}, $\theta_{ANC\text{-}A}$, $\theta_{ANC\text{-}B}$, and $\theta_{ANC\text{-}CB}$) and contemporary (θ_A, θ_B, and θ_C) effective population sizes, timing of divergence (T_{DIV1}, T_{DIV2}), timing of hybridisation (T_{HYB}), and contribution coefficient (γ_{HYB}). These six models were also evaluated either assuming symmetrical contemporary migration ($m_{I\text{-}J}$) among all demes (not shown on hypothetical scenarios).

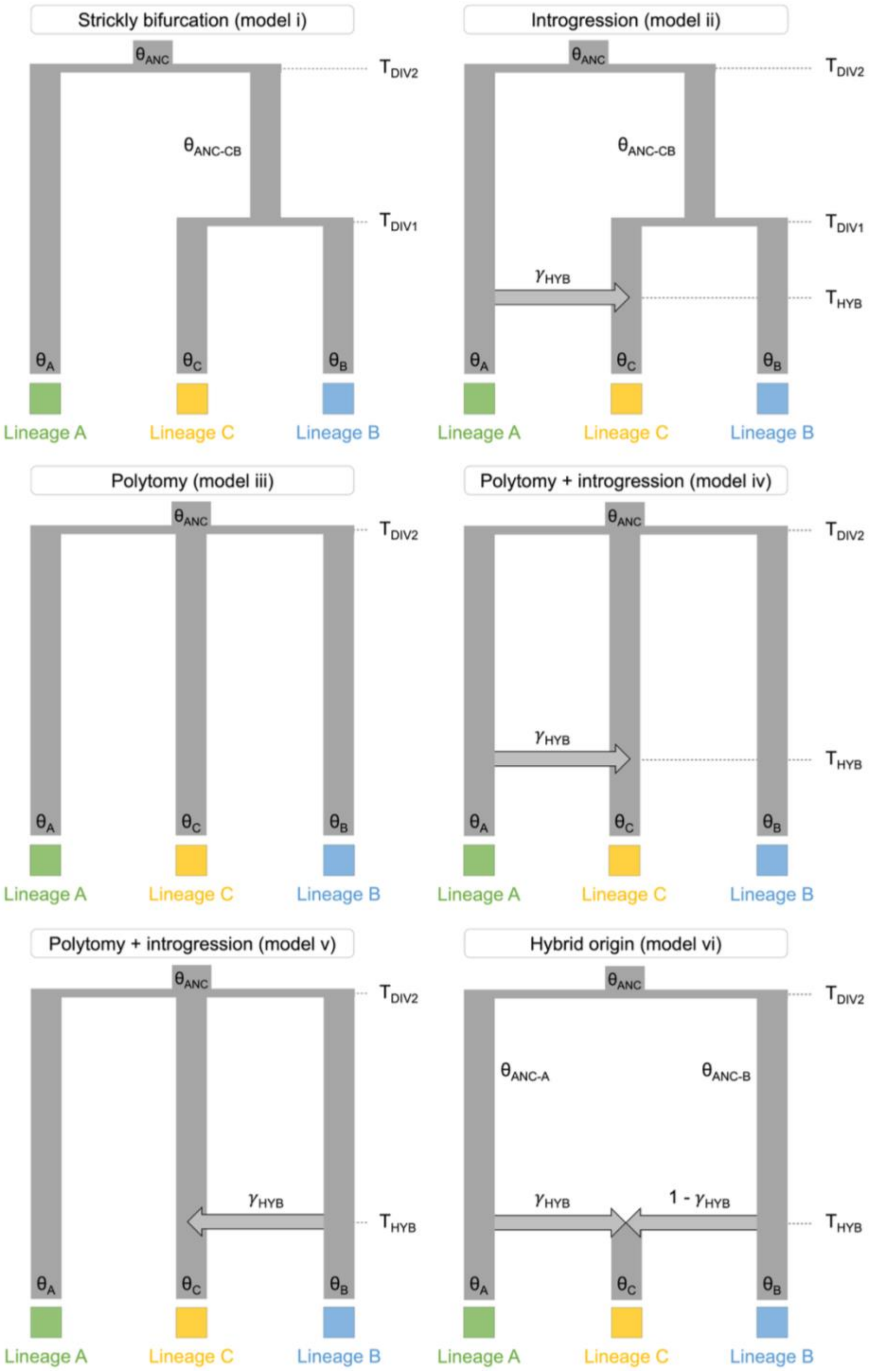

Figure S6. Phylogenetic relationships among individuals of the *Silvacalles instabilis* species complex, as inferred from the barcode region of the mitochondrial DNA (mtDNA) cytochrome c oxidase subunit I (COI) using IQ-TREE. Associations between mtDNA haplotypes and higher-genetic groups based on genome-wide nuclear data are mapped onto the mtDNA tree. Asterisks on the tree denote well-supported nodes (bootstrap value >85). Four individuals belonging to the sister species *S. nubilosus* were used as an outgroup. Colours as in Figure 1.

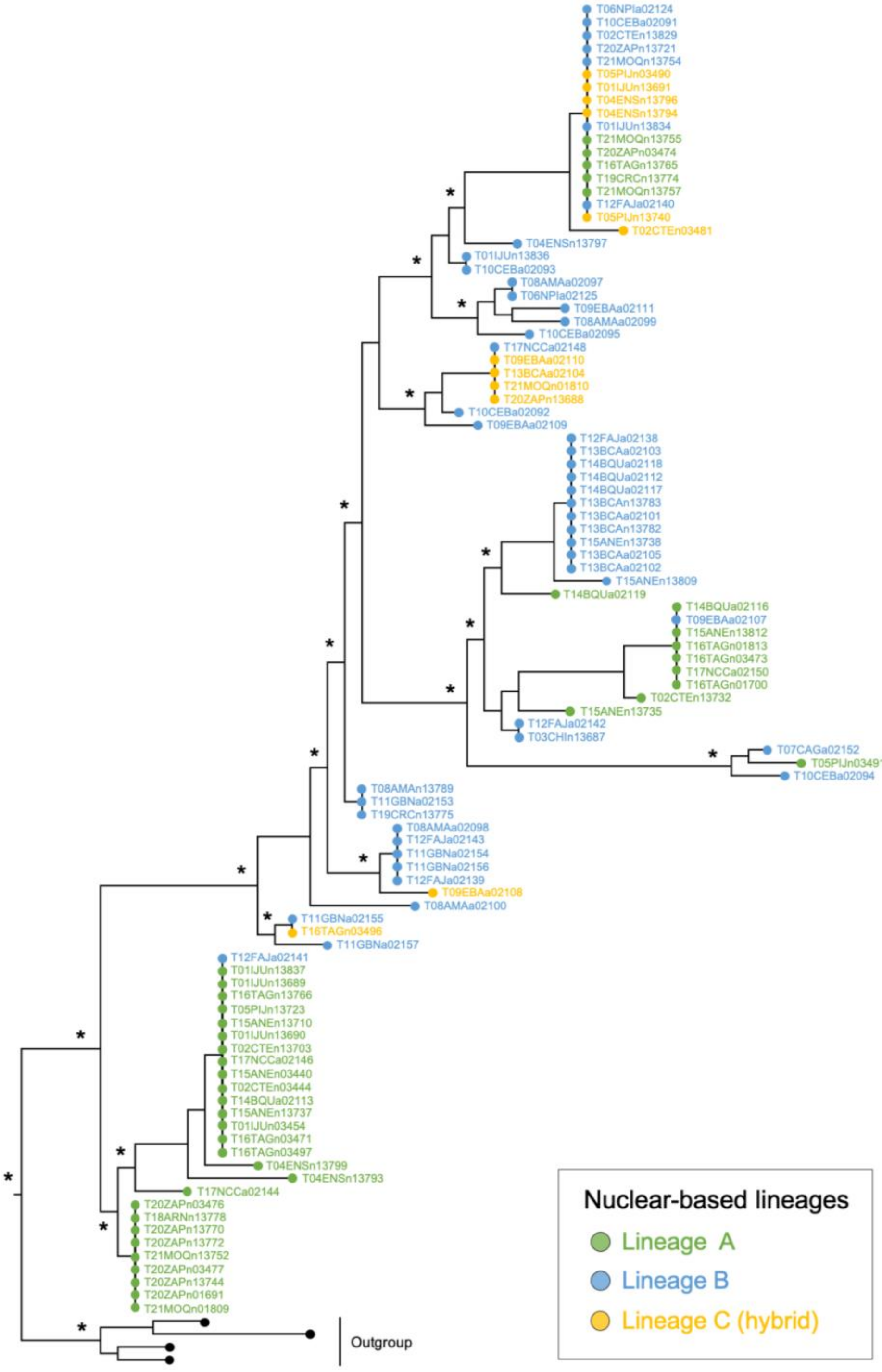

Figure S7. Mean (±SD) log probability of the data (LnPr(X|*K*); (left axes, open dots and error bars) and the magnitude of Δ*K* (right axes, black dots and continuous line) for each value of *K*-value estimated over 10 STRUCTURE runs for three different analyses including (a) all individuals from the *Silvacalles instabilis* species complex, (b) only individuals assigned to lineage A, and (c) only individuals assigned to lineage B (Figure 1).

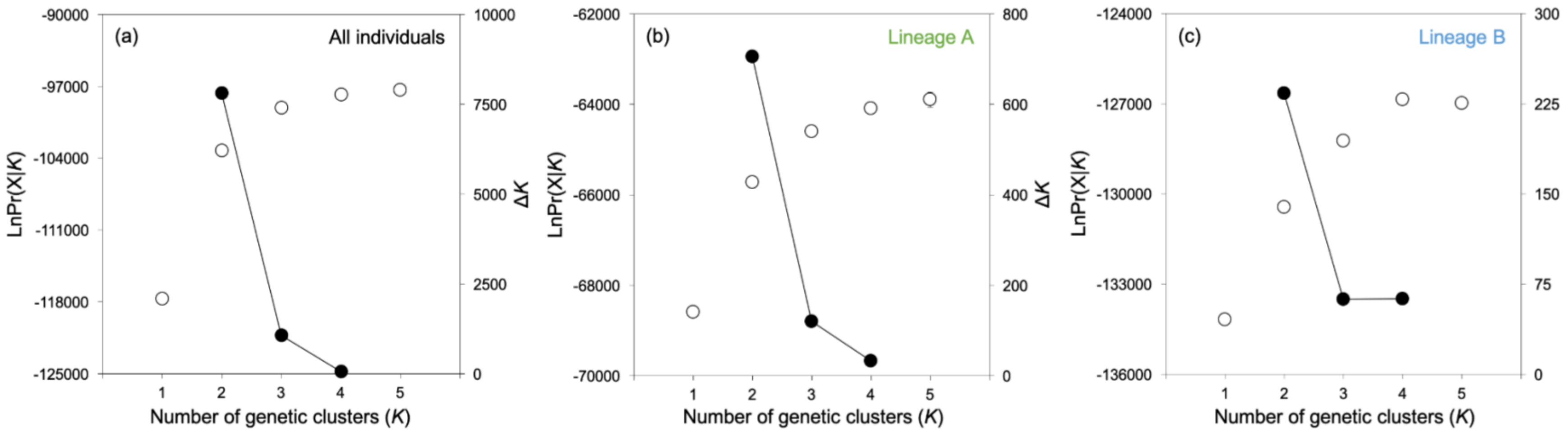

Figure S8. Geographical structuring of nuclear genomic variation within lineage A, as inferred with STRUCTURE (panel a) and a principal component analysis (PCA) (panel b). In panel (a), STRUCTURE bar plots depict the ancestry coefficients per individual assuming two and three ancestral populations (*K*). Thin vertical black lines separate individuals, which are partitioned into *K*-coloured segments representing the probability of belonging to a given ancestral population. Within the *K*=2 bar plot, black circles represent the level of genetic variability per individual as measured using the observed heterozygosity (H_O) parameter. Site codes as in Table S1. In panel (b), pie charts represent the position of each individual along the two first principal components (PCs) and their respective ancestry coefficients based on STRUCTURE results, assuming *K*=2. Size of pie charts represents individual-based H_O.

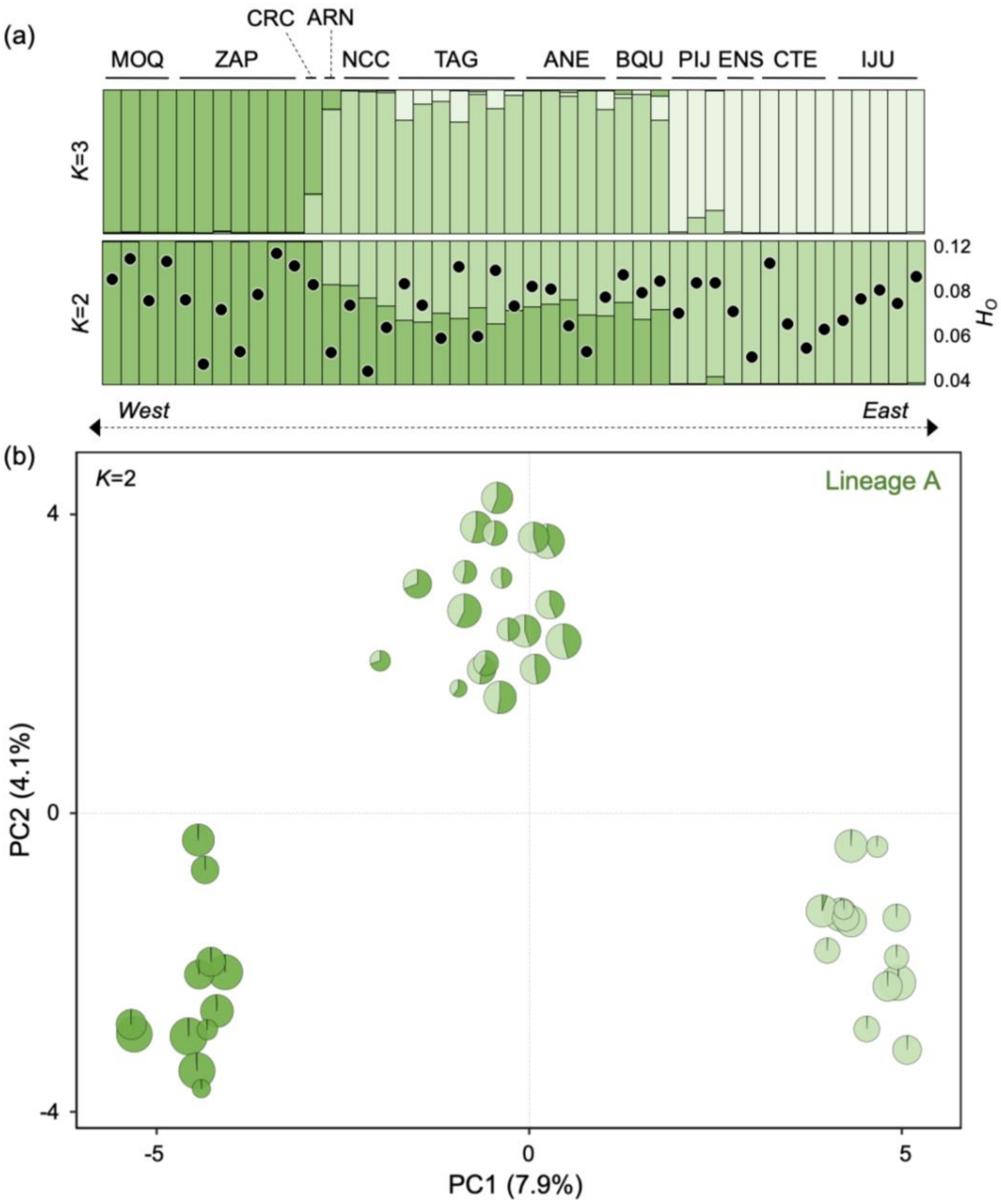

Figure S9. Geographical structuring of the genomic variation within lineage B, as inferred with STRUCTURE (panel a) and a principal component analysis (PCA) (panel b). In panel (a), the STRUCTURE bar plots depict the ancestry coefficients per individual assuming two and three ancestral populations (*K*). Thin vertical black lines separate individuals, which are partitioned into *K*-coloured segments representing the probability of belonging to a given ancestral population. Within the *K*=2 bar plot, black circles represent the level of genetic variability per individual as measured using the observed heterozygosity (H_O) parameter. Site codes as in Table S1. In panel (b), pie charts represent the position of each individual along the two first principal components (PCs) and their respective ancestry coefficients based on STRUCTURE results, assuming *K*=2. Size of pie charts represents individual-based H_O.

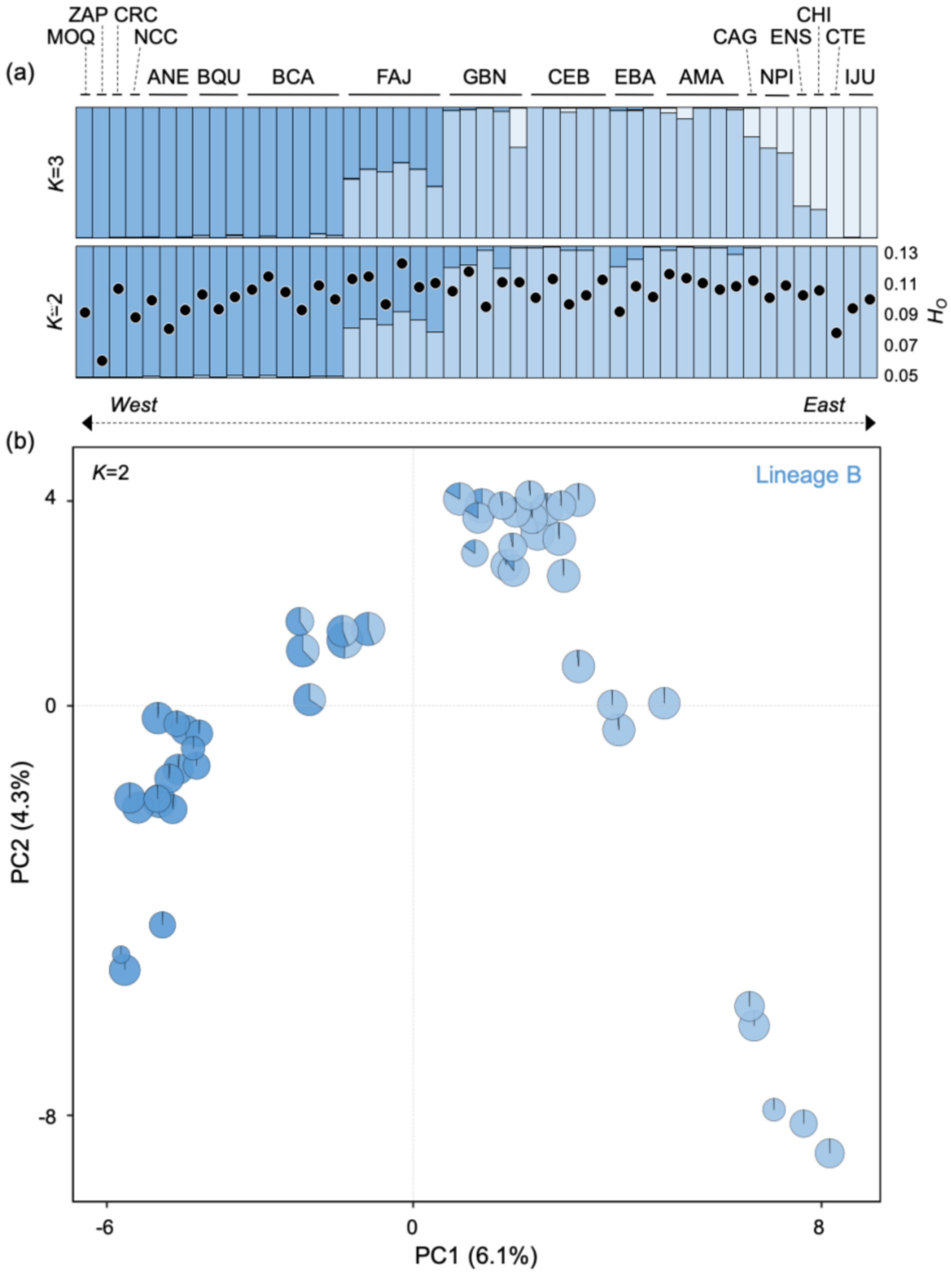

Figure S10. Observed heterozygosity (*H*o) across the higher-order genetic groups according to STRUCTURE results, assuming three ancestral populations (*K*=3; Figure S3). Letters below box plots indicate that differences between the respective groups are not statistically significant (*P*-value >0.05) after *post hoc* tests using both the Tukey and Wilcoxon methods.

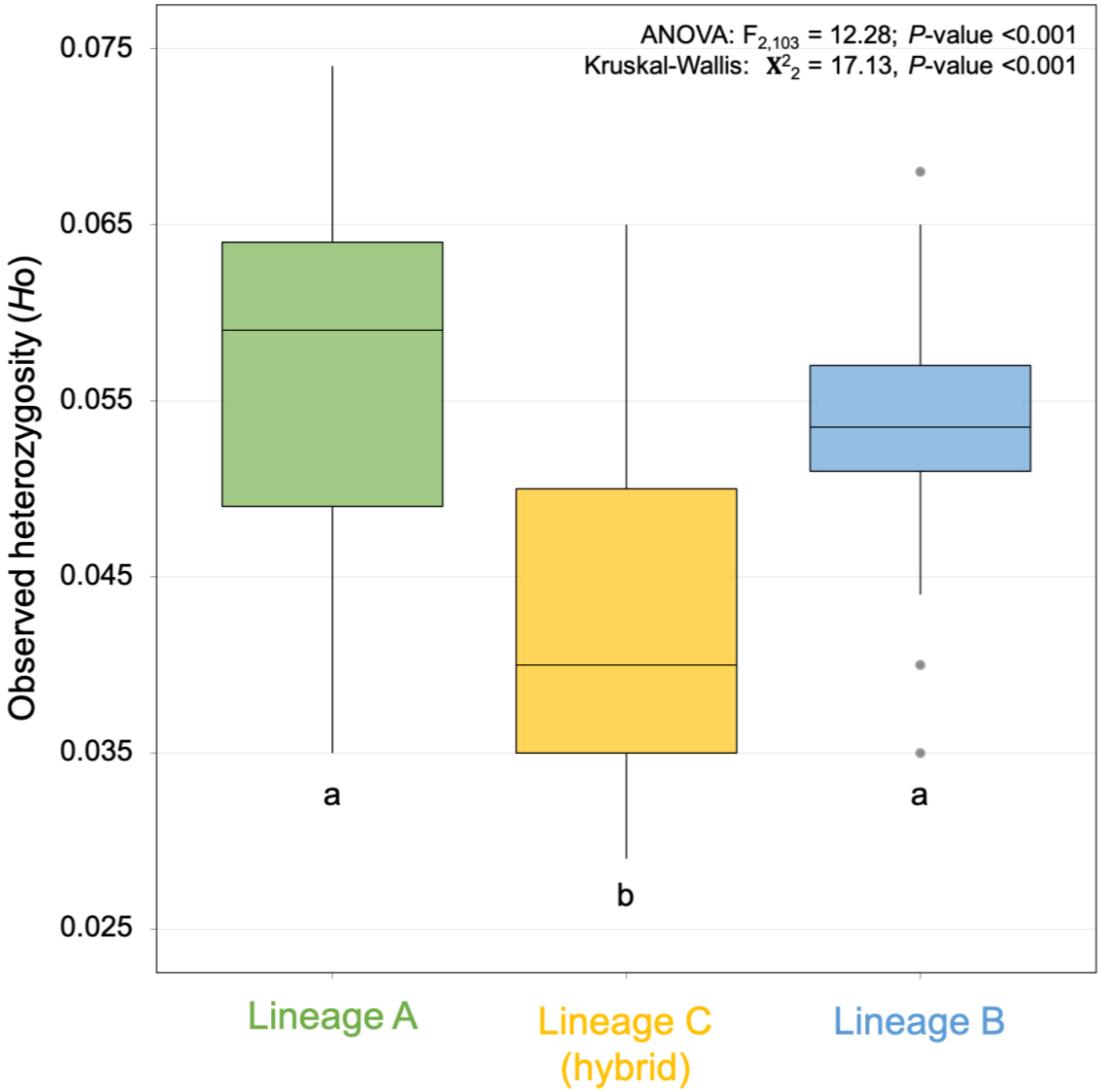

Figure S11. Individual-based simulations of the evolution of hybrid populations. (a) Triangle plot showing replicate trajectories under a scenario where the ancestry-informative markers (AIMs) were arrayed on 20 linkage groups (LG). Each grey line represents the trajectory of one hybrid population over time. The three highlighted trajectories in dark blue, light blue and red represent a random path, an outlier, and the path closest to the overall mean, respectively. The black dashed line indicates the mean trajectory across all replicates. The mean value of ${}_{i}He$ and Hb_{i} are shown for lineages A and B, and the F_1 generation. The mean of lineage C at the time of reaching mean parental ${}_{i}He$ (mean ${}_{i}He_{AB}$) is indicated by the yellow circle. (b) Density plot showing the time (in N_e generations) to reach ${}_{i}He_{AB}$ across five scenarios with varying degrees of linkage. The x-axis shows time (N_e generations), and the y-axis shows the density of replicate simulations. (c) Density plot of ancestry proportions for each replicate at the time it reached mean ${}_{i}He_{AB}$ for the same linkage scenarios, with ancestry proportion on the x-axis and density on the y-axis. (d) Triangle plots of bi-parental migration under the free recombination scenario with migration rates of m=0.005, 0.001, 0.0005 and 0.0001, with colour and axis schemes identical to (a). (e) Triangle plots of uni-parental migration at the same four migration rates, with colour and axis schemes identical to (a).

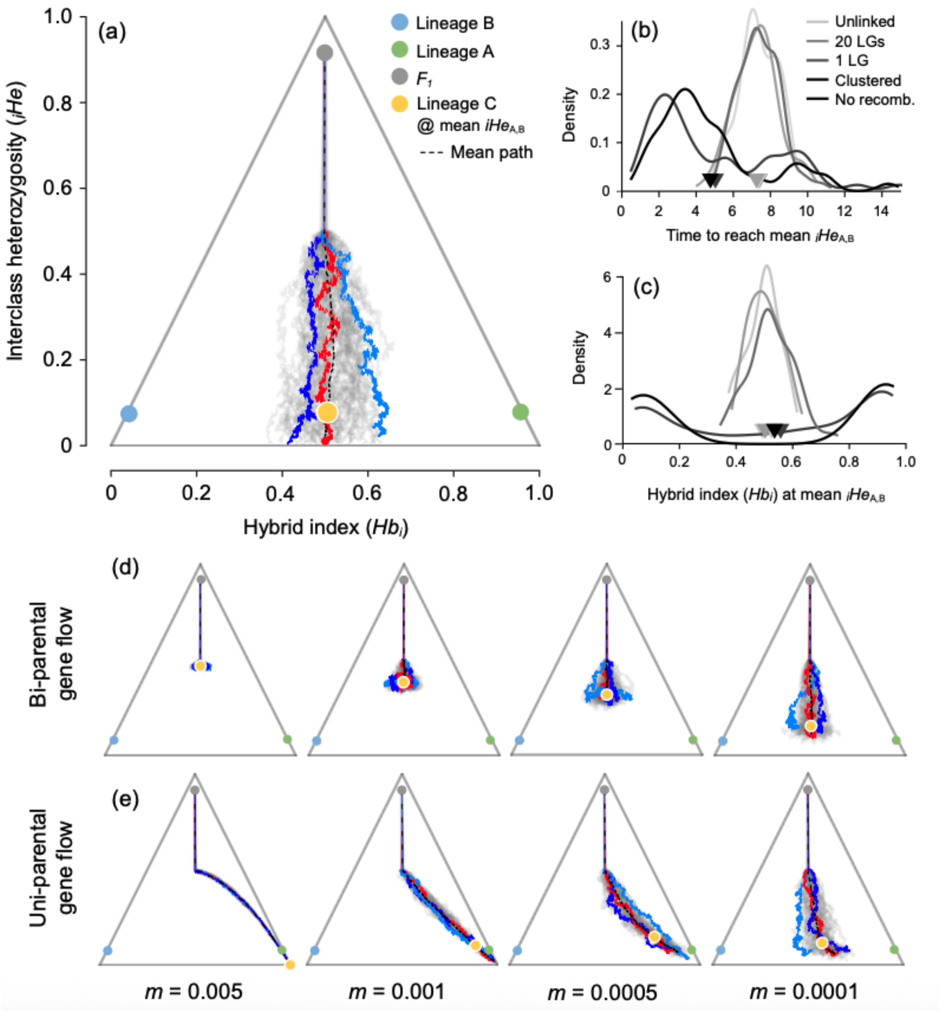

Figure S12. Triangle plots showing the joint distribution of ancestry (Hb_i) and interclass heterozygosity ($_iHe$) for 50 replicate hybrid populations (lineage C) over 15 N_e generations under varying linkage scenarios: (a) free recombination, (b) single chromosome, (c) tight linkage (0.5 cM), and (d) complete linkage (no recombination). Grey lines show population trajectories from the F_1 generation; mean lineage A (green circle), lineage B (blue circle), F_1 (grey circle), and mean of lineage C at the time of reaching mean parental $_iHe$ (mean $_iHe_{AB}$) (yellow circle). Highlighted trajectories (dark blue, light blue, red) represent a random path, an outlier, and the trajectory closest to the mean. The black dashed line shows the mean trajectory. Axes show mean hybrid index (x-axis) and mean interclass heterozygosity (y-axis).

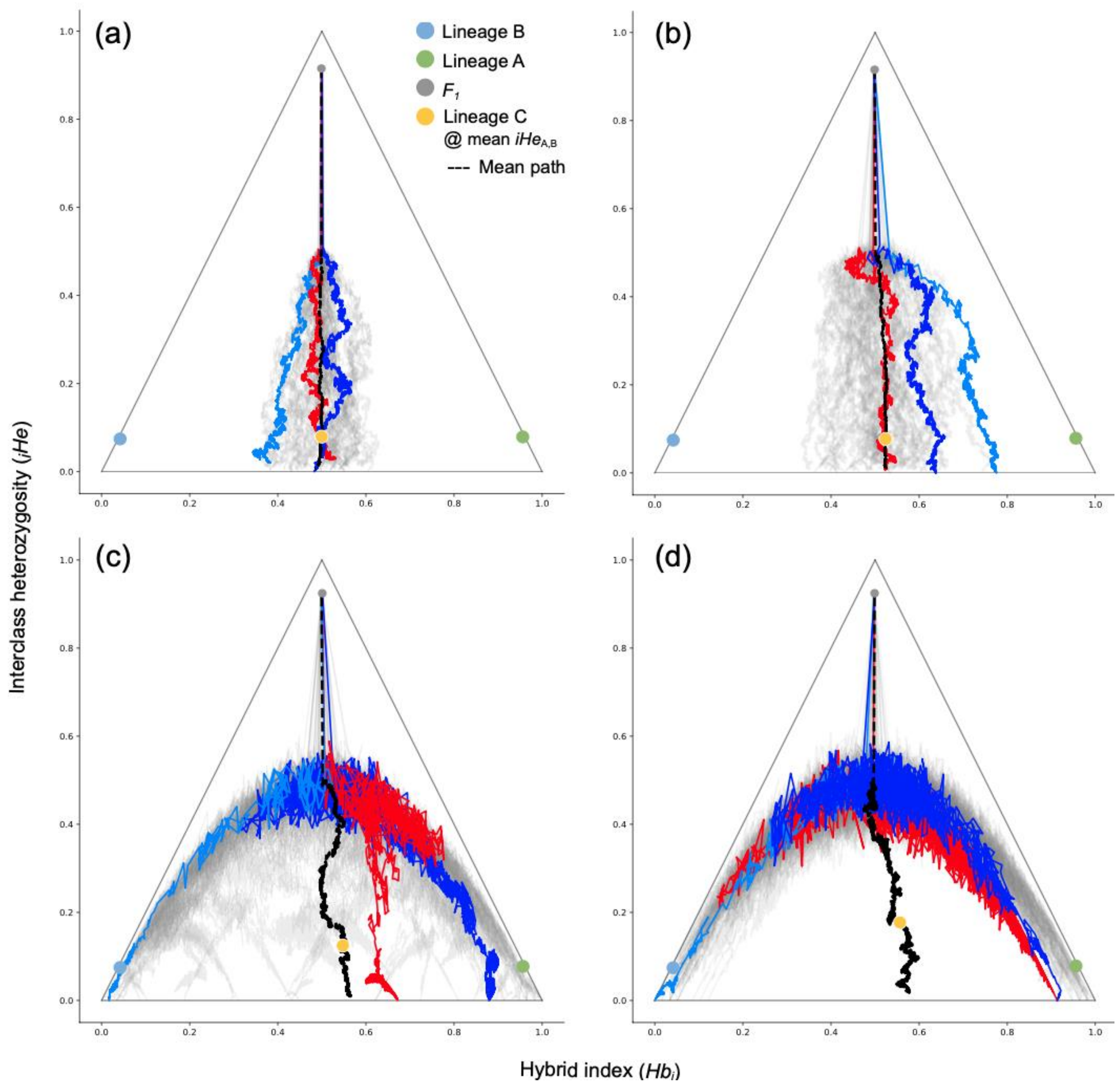

Figure S13. Structuring of genomic variation within hybrid lineage C with regard to one of the two parental lineages A and B, as inferred with a principal component analysis (PCA). Dashed and arrowed lines highlight west-east geographical structuring of genetic variation within lineages. For lineage C, individuals sampled in the westernmost sites (MOQ, ZAP) are highlighted with a hash symbol, with those sampled in the easternmost sites (ENS, CTE and IJU) being highlighted with an asterisk. As in Figure 1, the position of the F_1 individual between lineages B and C (EBA site) is identified with a black arrow. Colours as in Figure 1.

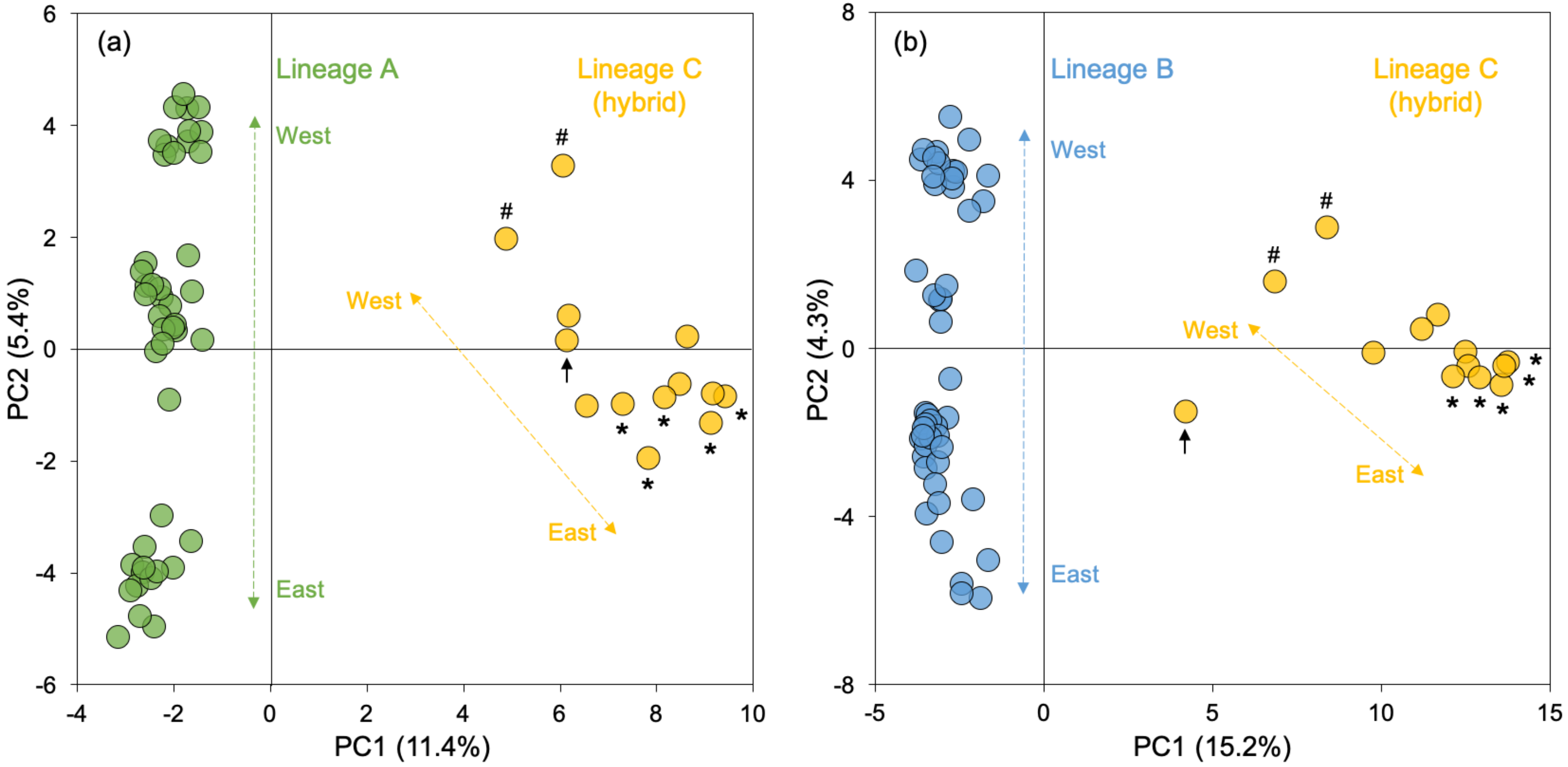

Figure S14. Allelic variation for the retained ancestry-informative markers (AIMs) across individuals from the lineage C and parental lineages A and B. AIMs correspond to those retained for the analyses testing for the presence of different hybrid classes (*i.e.*, F_1, F_2, backcrosses and later-generation hybrids) when all individuals from lineage C are compared against all individuals from lineages A and B, which are considered parentals. Individuals are ordered geographically within each lineage. Colours as in Figure 1.

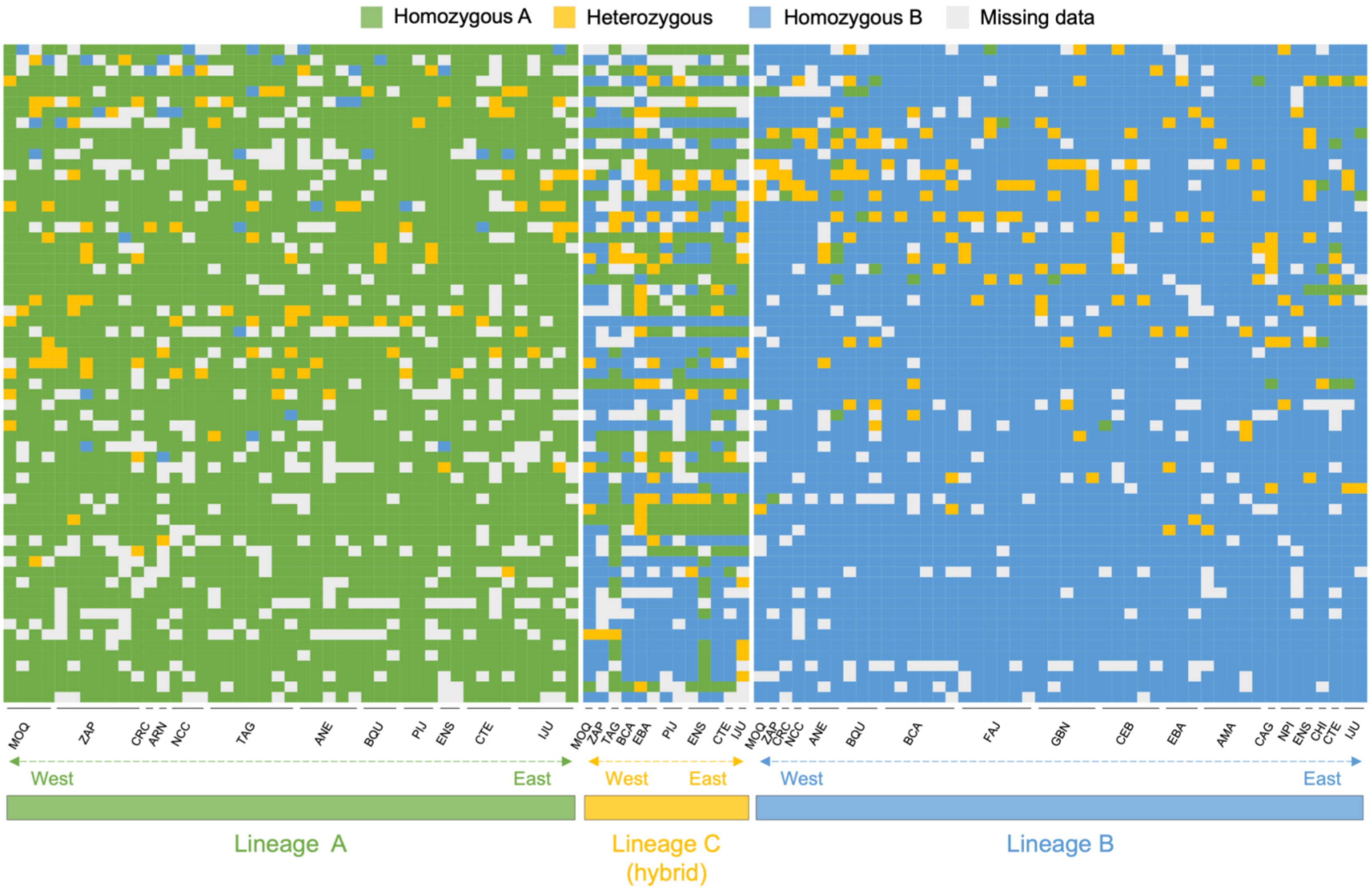

Supplement: Supplementary file 1 [file rspb.2026.1745.zip › rspb.2026.1745/rspb20261745_si_001.pdf]
